# Correlation between manganese dissolution and dynamic phase stability in spinel-based lithium-ion battery

**DOI:** 10.1038/s41467-019-12626-3

**Published:** 2019-10-17

**Authors:** Tongchao Liu, Alvin Dai, Jun Lu, Yifei Yuan, Yinguo Xiao, Lei Yu, Matthew Li, Jihyeon Gim, Lu Ma, Jiajie Liu, Chun Zhan, Luxi Li, Jiaxin Zheng, Yang Ren, Tianpin Wu, Reza Shahbazian-Yassar, Jianguo Wen, Feng Pan, Khalil Amine

**Affiliations:** 1 School of Advanced Materials, Peking University, Shenzhen Graduate School, 518055 Shenzhen, China; 2 Chemical Sciences and Engineering Division, Argonne National Laboratory, Argonne, IL 60439 USA; 3 Department of Mechanical and Industrial Engineering, University of Illinois at Chicago, Chicago, IL 60607 USA; 4 X‐ray Science Division, Argonne National Laboratory, Argonne, IL 60439 USA; 5 Center for Nanoscale Materials, Argonne National Laboratory, Argonne, IL 60439 USA; 6 Material Science and Engineering, Stanford University, Stanford, CA 94305 USA; 7 Institute for Research and Medical Consultations (IRMC), Imam Abdulrahman Bin Faisal University (IAU), Dammam, 34212 Saudi Arabia

**Keywords:** Batteries, Batteries

## Abstract

Historically long accepted to be the singular root cause of capacity fading, transition metal dissolution has been reported to severely degrade the anode. However, its impact on the cathode behavior remains poorly understood. Here we show the correlation between capacity fading and phase/surface stability of an LiMn_2_O_4_ cathode. It is revealed that a combination of structural transformation and transition metal dissolution dominates the cathode capacity fading. LiMn_2_O_4_ exhibits irreversible phase transitions driven by manganese(III) disproportionation and Jahn-Teller distortion, which in conjunction with particle cracks results in serious manganese dissolution. Meanwhile, fast manganese dissolution in turn triggers irreversible structural evolution, and as such, forms a detrimental cycle constantly consuming active cathode components. Furthermore, lithium-rich LiMn_2_O_4_ with lithium/manganese disorder and surface reconstruction could effectively suppress the irreversible phase transition and manganese dissolution. These findings close the loop of understanding capacity fading mechanisms and allow for development of longer life batteries.

## Abstract (web summary)

To unlock the potential of Mn-based cathode materials, the fast capacity fading process has to be first understood. Here the authors utilize advanced characterization techniques to look at a spinel LiMn_2_O_4_ system, revealing that a combination of irreversible structural transformations and Mn dissolution takes responsibility.

## Introduction

Performance improvement of cathode materials represent one of the most critical technological challenges for lithium ion batteries (LIBs)^1–5^, as existing cathode materials exhibit underachieved cycling stability and severe capacity loss^6–10^. Cathode material stability is predominately attributable to two factors: bulk structural stability and surface chemical stability^11–14^. In current commercial LIBs (lithium/transition metal (TM) oxides or polyanionic compounds), TM ions function as redox centers that facilitate rapid electron exchange and accompanying reversible structural evolution^15–17^. Thus, their effects for bulk structural stability and surface chemical stability are crucial to the electrochemical performance of LIBs. Unfortunately, almost all cathode TM ions have been observed to suffer from pronounced dissolution^18–20^ that subsequently induces a negative effect on the anode. Conventionally this has been considered as the root cause of capacity fading for batteries^21–23^. However, despite thorough investigations into TM migration mechanisms on cathode surfaces^24^, structural evolution of cathode materials undergoing TM dissolution and their interrelationships have not been clear. TM loss from the cathode could very likely lead to irreversible structural transformation, which potentially produces adverse effects on cathode structural stability. These are significant factors for cathode cycling performance improvement, but currently are still not completely understood.

Spinel lithium manganite (LiMn_2_O_4_) is commonly chosen as the model material to study capacity loss due to its pronounced Mn(II) ion dissolution^25,26^. Although accepted as the root cause of capacity fading^27^, observed Mn dissolution is far less than capacity loss found in electrochemical tests. This demonstrates that capacity loss does not result from active redox element loss, but rather its subsequent anode and cathode impact. Mn deposition on anode, a topic previously well studied^28–30^, gradually interferes with lithium intercalation and increases anode impedance, which definitively shortens battery cycle life. However, as the source of Mn, spinel cathode LiMn_2_O_4_ undergoing Mn dissolution not only causes active surface ion loss but also potentially triggers irreversible structural damage. Even though spinel phase transition behaviors have been reported previously^31–33^, most of them were independently investigated without involving Mn dissolution, while the interaction of Mn dissolution and phase transition is yet to be understood. Moreover, the discrepancies that exist between Mn dissolution and capacity fading of spinel cannot be reasonably explained with currently accepted Mn(III) disproportionation mechanisms^34,35^. On the one hand, although Mn dissolution theoretically decreases at charging potentials above 4 V with decreased Mn(III) content, it has been observed to dramatically increase in experiments reported by Jang et al.^19^. On the other hand, Aurbach et al.^36^ had recently reported that Mn(III) was the dominant soluble Mn ion species in electrolyte solutions, rather than Mn(II) generated from Mn(III) disproportionation. With all things considered, previous conclusions that Mn dissolution or phase transformations solely contribute to LiMn_2_O_4_ capacity decay are incomprehensive and should be revisited. Understanding intrinsic cathode stability and the relationship between structural evolution and Mn dissolution is essential to solve capacity fading and stability issues.

In this work, we aim to address the aforementioned knowledge gap between Mn dissolution, structural evolution, and their consequence. We employ spinel LiMn_2_O_4_ as a model system to explicitly demonstrate what cathode structural evolutions are involved in charge/discharge processes and how structural evolutions interact with Mn dissolution. Li-rich Li_1+*x*_Mn_2−*x*_O_4_ is used as a control sample to explore underlying Mn dissolution mechanisms and structural stability, as excess Li in spinel structures have demonstrated beneficial inhibition of Mn dissolution and have improved cycle performances previously^37,38^. Through advanced X-ray techniques, including X-ray diffraction (XRD), X-ray absorption spectroscopy (XAS), and X-ray fluorescence (XRF), together with scanning transmission electron microscopy (STEM), it is evident that LiMn_2_O_4_ suffers from severe irreversible phase evolutions. These transitions produce an unexpected, soluble Mn_3_O_4_ phase driven by Mn(III) disproportionation during charging, an over-lithiated Li_2_Mn_2_O_4_ at high discharge potential (3.4 V), and particle cracks during cycling. These side reactions are the predominate reasons for fast Mn dissolution in LiMn_2_O_4_. Meanwhile, synergy between fast Mn dissolution and Jahn–Teller distortion continuously triggers generation of over-lithiated surface Li_2_Mn_2_O_4_, which in turn facilitates irreversible phase transformation and particle cracks. These unprecedented findings unambiguously enable us to better understand the mechanisms of spinel LiMn_2_O_4_ capacity fading: continuous irreversible phase transition and Mn dissolution form a vicious cycle that constantly consumes the capacity of LiMn_2_O_4_. It is found that the atomic structural advantages of Li-rich LiMn_2_O_4_ (Li/Mn disordering and surface reconstruction) can effectively mitigate irreversible phase transitions, which suppress Mn dissolution and formation of particle cracks. The insight into cathode capacity decay can serve as design principles to facilitate future discovery of improved, structurally stable cathode materials in LIBs.

## Results

### Structural evolution analysis

Stoichiometric LiMn_2_O_4_ (defined as LMO) and lithium-rich Li_1.09_Mn_1.91_O_4_ (defined as LR-LMO) samples were synthesized as described in the Methods section. In addition, compositions obtained from chemical and morphological analyses are provided in Supplementary Information (Supplementary Table 1, Supplementary Fig. 1), while electrochemical performances of LMO and LR-LMO tested in both half-cell and full-cell configurations can also be found in the Methods section and Supplementary Information (Supplementary Figs. 2 and 3). The charge–discharge curves of LMO and LR-LMO at 0.1C showed expected reversible capacities of 123 and 117 mAh g^−1^ for materials with these compositions^39,40^, respectively. However, the charge/discharge curves for these two materials were rather different. LMO presented two voltage plateaus found in both charge and discharge stages, while LR-LMO voltage profiles maintained a steep slope throughout all cycles. A much more obvious distinction between the plateau shapes can be observed from differential capacity vs. voltage (d*Q*/d*V* vs. *V*), as shown in Supplementary Fig. 2b. The d*Q*/d*V* curve of LMO exhibits two sharp peaks at 4.05 and 4.15 V that correspond to two distinct phase transition processes. Although appearing at a similar voltage as LMO, the d*Q*/d*V* peaks of LR-LMO broaden significantly, indicating a smooth conversion between the two-phase transitions.

**Fig. 1:**
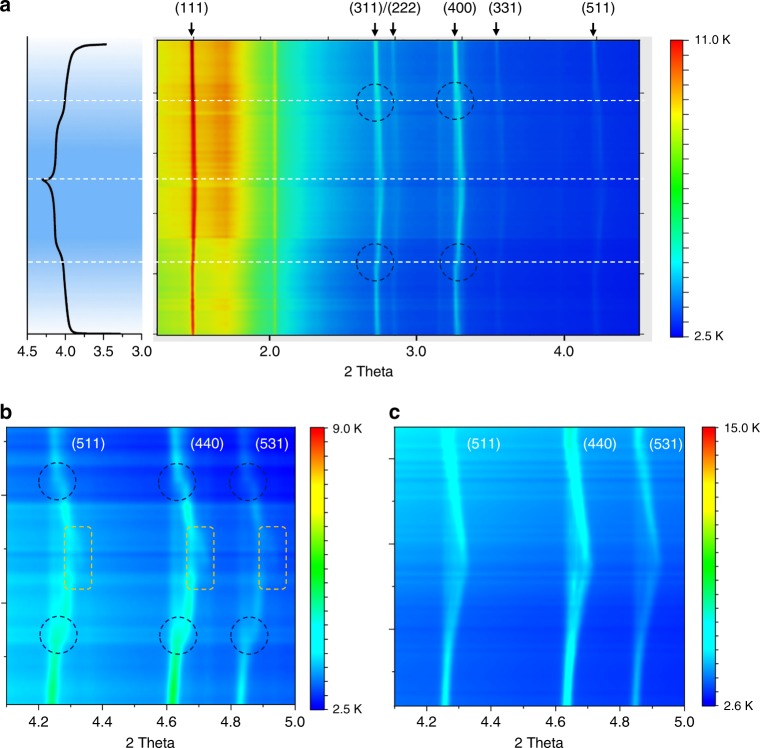
In situ synchrotron HEXRD characterization on the structural evolution of LMO and LR-LMO during the first charge/discharge. **a** The in situ XRD curve of the first charge and discharge process and first cycle electrochemical profile for LMO; **b** enlarged figure of Bragg peaks (511), (440), and (531) for LMO; **c** enlarged figure of Bragg peaks (511), (440), and (531) for LR-LMO

Operando high-energy XRD (HEXRD)^41^ was carried out to directly monitor cathode structural evolutions during charge and discharge. The schematic for in situ synchrotron HEXRD set-up and the home-made coin cell are presented in Supplementary Fig. 4 (experimental details can be found in the Methods section). The results are also respectively presented as contour (Fig. 1a, Supplementary Fig. 5) and waterfall plots (Supplementary Figs. 6 and 7). Interestingly, in situ XRD results of the two samples correlated well with their electrochemical behaviors and also directly reflected structural evolution discrepancies. Obvious differences between the two XRD patterns appeared at 4.05 V, where LMO exhibited a clear phase transition turning point (Fig. 1a, navy dashed circle), while LR-LMO evolved smoothly and exhibited single-phase solid-solution type insertion/deinsertion behavior (Supplementary Fig. 5). In order to clarify data presentation, three brag peaks at (511), (440), and (531) of LMO and LR-LMO are magnified and shown in Fig. 1b, c, respectively. Figure 1b shows that LMO exhibits two-phase transition stages during the charge/discharge process (navy dashed circle), consistent with the two peaks in its d*Q*/d*V* curve. In the first stage with less than 40% lithium extraction (charge), the three peaks visibly shift towards high diffraction angles (2*θ*) due to lattice parameters decreases during lithium removal. Once lithium extraction exceeds 40% (around 4.05 V, Li_*x*_Mn_2_O_4_, *x* = 0.6), the phase transition rapidly enters into the second stage and spinel Li_*x*_Mn_2_O_4_ begins to convert to cubic *λ*-MnO_2_^42,43^. Particularly noticeable, three new-appearing peaks with fairly low intensity are visible in the LMO near the end of charge (Fig. 1b, yellow dashed rectangle and Supplementary Fig. 8a), located on the right side of (511), (440), and (531), respectively. These newly generated peaks would not completely disappear even at the fully lithiated stage (end of discharge, 3.4 V). However, peak intensity apparently weakens during discharge, clearly suggesting this new phase formed during charge seems to be irreversible. Surprisingly, a much closer look at the Bragg peaks ((511), (440), and (531)) at the end of discharge shows noticeable deviation from the Gaussian function and could refer to generation of an additional phase (Supplementary Fig. 8a). Although it is difficult to identify these two phases solely based on in situ XRD data (due to relatively low intensity), such irreversible phase transition observed, even just during the first cycle, will be undoubtedly responsible for cathode stability. This is further supported by the similar test on the LR-LMO, which exhibits better structural reversibility during the charge/discharge process. Figure 1c shows that a single-phase reaction smoothly transfers to a two-phase reaction following a solid-solution behavior in a LR-LMO sample during the removal of lithium. Upon discharge, the XRD pattern entirely converts back to that of the pristine phase via the converse sequence of structure transitions. Additionally, the absence of new peaks even at high potentials proves that the irreversible phase transition is mitigated in LR-LMO (Fig. 1c, Supplementary Fig. 8b).

### Quantitative analysis and observation of LMO phase evolution

**Fig. 2:**
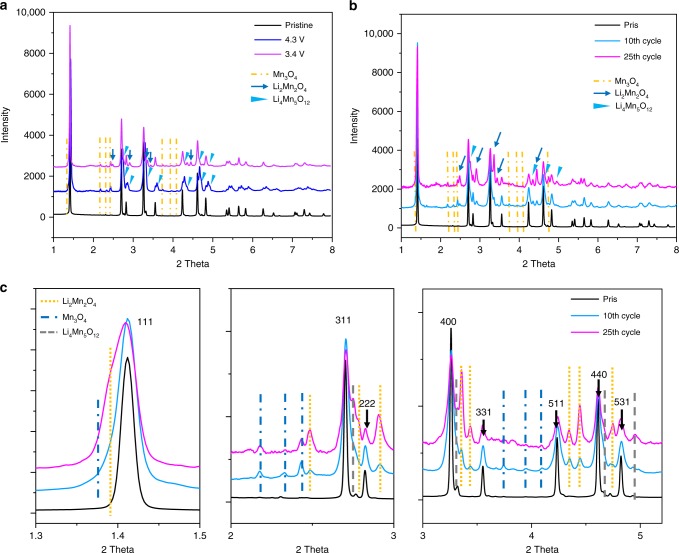
Qualitative structural analysis of LMO with different potentials and cycles. **a** Ex situ XRD pattern of LMO at different potentials. **b** Ex situ XRD patterns of LMO at different cycles. **c** The enlarged figures of the red dashed rectangles of **b**

To further grasp insights into the above-observed irreversible LMO phase transition, ex situ XRD with high structural resolution was applied to precisely identify phase composition at different potentials. Figure 2a clearly shows that, in addition to the spinel LMO Bragg peaks, a series of small peaks appear in the XRD pattern in the fully charged LMO electrode (4.3 V). By utilizing in situ XRD, these additional peaks can be readily indexed to a spinel Li_4_Mn_5_O_12_ phase, which confirms that an unexpected phase transition occurs during first charge. In addition, a small amount of spinel-like Mn_3_O_4_ structure can be surprisingly identified in XRD patterns (Fig. 2a). Mn_3_O_4_, which has an average oxide state of 8/3, certainly cannot be produced from an electrochemical reaction during the oxidation process (charge)^44,45^. Considering the presence of Mn(II) in Mn_3_O_4_ and Mn(VI) in Li_4_Mn_5_O_12_, these phase transitions are very likely associated with the Mn(III) disproportionation reaction, in which Mn(III) transfers to Mn(II) and Mn(VI). Thus, this chemical reaction can be descripted as in Eq. (1), where LiMn_2_O_4_ transforms to Li_4_Mn_5_O_12_ and Mn_3_O_4_ under the drive of Mn(III) disproportionation. For easier understanding, this equation can also be simplified into Eq. (2) to clearly expound the Mn(III) disproportionation reaction.

1 $$\mathrm{LiMn}_{2}\mathrm{O}_{4} \to \frac{1}{4}\mathrm {Li}_{4}\mathrm{Mn}_{5}\mathrm{O}_{12} + \frac{1}{4}\mathrm {Mn}_{3}\mathrm{O}_{4}$$

2 $$	\frac{1}{2}{\mathrm{Mn}}^{3 + }\left( {\mathrm{LiMn}}_2{\mathrm{O}}_{4} \right){\,} {\mathop{\longrightarrow}\limits^{{\mathrm{disproportionation}}\,{\mathrm{reaction}}}}\, \frac{1}{4}{\mathrm{{Mn}}}^{4 + }\left( {\mathrm{Li}}_{4}{\mathrm{Mn}}_{5}{\mathrm{O}}_{12} \right) \\ 	\hskip 12pt + \frac{1}{4}{\mathrm{Mn}}^{2 + }\left( {\mathrm{Mn}}_{3}{\mathrm{O}}_{4} \right)$$

To the best of our knowledge, this is the first time that Mn(III) disproportionation and subsequent phase transitions caused by charged states have been tracked by solid experimental evidence. Interestingly, Mn_3_O_4_, with a low Mn oxide state, has proven to be soluble by the dissolution experiment of Mn_3_O_4_ in electrolyte under steady conditions (Supplementary Fig. 9), where the increased Mn content was clearly detected in electrolyte with prolonged time periods. The presence of soluble Mn_3_O_4_ aptly explains the previous phenomenon reported by Jang et al.^19^ that though the average Mn oxide state increases with higher potentials, accelerated Mn dissolution has been observed during charge. Moreover, these new insights highlight the significant effect of structural stability on cathode materials.

Of particular interest is the reversibility of the Mn(III) disproportionation induced phase transition. As shown in Fig. 2a, the characteristic peaks of Mn_3_O_4_ and Li_4_Mn_5_O_12_ still clearly appear in the XRD pattern at full lithiation (3.4 V, discharge), which confirms that these phase transitions are irreversible. Undoubtedly, these irreversible phase transitions would predominately contribute to the fast capacity fading of LMO. In addition to the structural peaks mentioned above, some weak Li_2_Mn_2_O_4_ that usually form below 2.9 V upon Mn(VI) shift to Mn(III), are unexpectedly detected at a fully lithiated potential of 3.4 V (Fig. 2a, Supplementary Fig. 10). This new Li_2_Mn_2_O_4_ phase was also confirmed with Mn valance decreases observed from ex situ XAS measurements, where the main edge shifted to lower energy upon discharge (Supplementary Fig. 11). Its appearance provides a rational explanation for peak deviations observed in the in situ XRD measurement at the end of discharge. Further confirmations, derived from the XRD patterns of 10 and 25 cycles, display the increased signals of Li_4_Mn_5_O_12_ and Li_2_Mn_2_O_4_, especially over-lithiated Li_2_Mn_2_O_4_, which indicates that these unexpected phase transformations continuously increase during repeated cycling. Unchanged Mn_3_O_4_ should be attributed to the dynamic equilibrium of its dissolution and regeneration. Of particular interest is the phase transition from spinel LiMn_2_O_4_ to tetragonal Li_2_Mn_2_O_4_, which is thoroughly discarded due to poor cycling stability at the initial stages of LIBs^46,47^. Based on our quantitative investigation (Supplementary Fig. 12), over 20% and 30% Li_2_Mn_2_O_4_ can be respectively calculated from structural refinement of the XRD patterns after 25 and 50 cycles, which suggests that the role of Li_2_Mn_2_O_4_ cannot be neglected during repeated charge/discharge. This phase transition process is formed from synergetic Jahn–Teller distortion effects^48,49^ that cause large anisotropic volume changes (16%) when going from cubic LiMn_2_O_4_ (cell parameter, *a* *=* *c* *=* 8.24 Å) to tetragonal Li_2_Mn_2_O_4_ (cell parameter, *a* *=* 8.01 Å, *c* *=* 9.27 Å), as well as subsequent severe structural damage in the active materials^50^. Thus, such large phase transitions with large anisotropic volume changes potentially trigger severe particle damage.

**Fig. 3:**
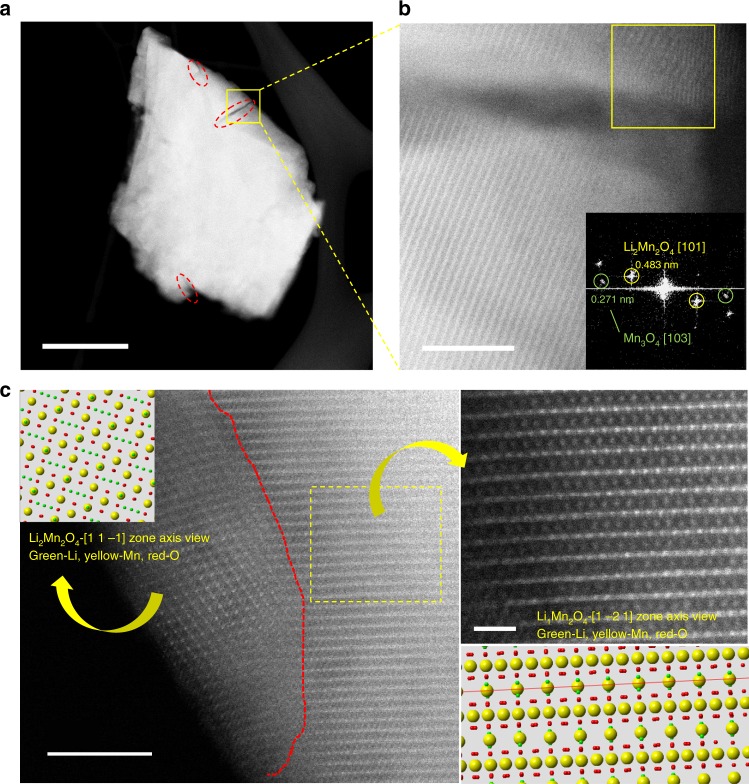
Visual observations of particle morphology and phase distribution from STEM-HAADF. **a** The STEM imaging of LMO particle after 25 cycles. **b** The high-resolution STEM imaging for the crack of **a**. **c** Visual atomistic-level observation of detailed structure and atomic occupancy for cycled LMO sample. Scale bars, 200 nm (**a**); 5 nm (**b**, **c**); 1 nm (enlarged figure on the right side of **c**)

Visual observations with TEM and EELS were conducted to further analyze particle morphology changes and detail phase distribution of LMO. Figure 3a and Supplementary Figure 13 certify that some cracks appear on the surface and extend more than 100 nm into the LMO particles after only 25 cycles. High-resolution TEM (Fig. 3b) indicates that both Li_2_Mn_2_O_4_ and Mn_3_O_4_ phases are distributed around particle surface cracks, which is highly consistent with the aforementioned XRD results. Atomic patterns observed with aberration-corrected STEM (Fig. 3c) further confirmed that the targeted particle surface structure deviates from the bulk structure, and exactly matches with that of Li_2_Mn_2_O_4_. In addition, EELS spectra show dramatic changes in both Mn-*L* and O-*K* edges from particle bulk to surface cracks. The peaks of Mn-*L*_3_ and Mn-*L*_2_ (Supplementary Fig. 14) exhibited a visible chemical shift towards lower energy loss, which directly indicated Mn valence decrease at surface cracks. Meanwhile, EELS O-*K* edge of the region around the crack matches well with that of Mn_3_O_4_ reported in earlier literature^51^. The EELS result certified the appearance of Mn_3_O_4_ on the side of cracks in cycled areas of LiMn_2_O_4_. Thus, the cracks should be attributed to huge cell parameter/strain changes and irreversible phase transitions. Accordingly, these cracks continuously grow with constant phase transformations and eventually crush the entire particle, which definitively aggravates capacity fading. Thus, by using a combination of in situ/ex situ XRD and TEM, we are able to represent an overall pattern of LiMn_2_O_4_ irreversible structure evolution: In the initial charge process, LiMn_2_O_4_ suffers from an irreversible phase transition driven by Mn(III) disproportionation, which consequently generates soluble Mn_3_O_4_ and irreversible Li_4_Mn_5_O_12_; upon discharge, Li_2_Mn_2_O_4_ is generated under the Jahn–Teller distortion at the end of lithiation, and continuously increases with cycling, resulting in some LMO particle cracks.

Compared to LMO, LR-LMO exhibits better structural stability during the charge/discharge process. Supplementary Figure 15 shows that the structure of LR-LMO still remains pure without any additional phase generation during the first charge/discharge process. Even after 25 cycles, almost no Mn_3_O_4_, Li_4_Mn_5_O_12_, and Li_2_Mn_2_O_4_ structures can be detected in the XRD pattern (Supplementary Fig. 16). TEM was also conducted to monitor the morphology change of LR-LMO after cycling. As shown in Supplementary Fig. 17, no crack is detected on the particle after 25 cycles. Therefore, Li enrichment can be considered an important strategy for improving structural stability and worthwhile to investigate further.

### Relationship between structure stability and capacity fading

According to previous works^21,52^, spinel LMO capacity fading is believed to be highly associated with Mn dissolution and its subsequent anode deposition during the cycling process. However, the effect of cathode structural stability on spinel LMO capacity fading has not been thoroughly investigated before. In lieu of simultaneous, irreversible phase transition and pronounced Mn dissolution during charge/discharge processes, some key issues should be considered further: (1) What is the relationship between Mn dissolution and structural evolution? (2) How do these reactions affect capacity fading?

**Fig. 4:**
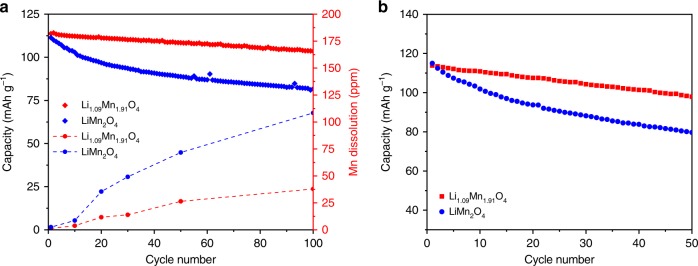
Mn dissolution analysis and cycling performances in half-cell and full cell. **a** Cycle performance of Li/LiMn_2_O_4_ half-cell (point curve) and concentration of Mn deposited on Li anodes harvested after different charge–discharge cycles (point-line curve). **b** The cycle performance was tested in Graphite/LiMn_2_O_4_ full cell (point curve) at 0.1C rate

To quantitatively investigate the degrees of Mn dissolution, inductively coupled plasma-atomic emission spectrometry (ICP-AES) and a synchrotron XRF were used to quantify the amount of Mn deposited on the anode in half cells and full cells. Figure 4a shows that both LMO and LR-LMO exhibit an increased deposition of Mn on the anode during half-cell cycling. Nevertheless, LMO still shows more severe Mn dissolution than LR-LMO at identical cycle rates. By the end of 100 cycles, the concentration of Mn for LMO reaches up to about 110 ppm while that of LR-LMO remains under 38 ppm. For full-cell graphite anodes, XRF results (Supplementary Figs. 18 and 19) show that Mn absorption intensity on graphite electrode for LR-LMO is three times less than that of LMO after 50 cycles, indicating that Mn dissolution and deposition of LR-LMO is far less than LMO. Cycling performance comparisons of these materials, shown in Fig. 4a, clearly indicate continuous capacity fading up to 85% of the initial value for LMO after 100 cycles. In contrast, the capacity of LR-LMO still preserves 93% of initial capacity after 100 cycles, exhibiting greatly improved cycling performance. Application of LR-LMO also shows greater full-cell cycling advantages; LR-LMO retained 86% of initial capacity, while LMO only retained 69% after 50 cycles (Fig. 4b).

**Fig. 5:**
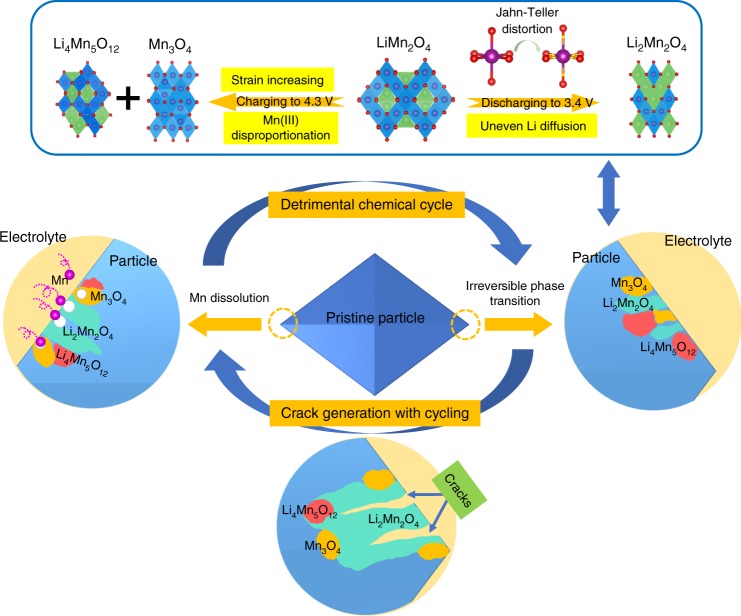
Schematic of the vicious cycle with Mn dissolution and irreversible phase transition, as well as crack generation with cycling

Based on the comparisons found in structural analysis and Mn dissolution measurements between LMO and LR-LMO, we can conclude that irreversible structural transformations definitively accelerate Mn dissolution. Full understanding of the LMO capacity fading mechanism is summarized in Fig. 5: soluble Mn_3_O_4_, an over-lithiated Li_2_Mn_2_O_4_ phase with full Mn(III), and particle cracks from cycling are the predominate reasons for accelerated Mn dissolution in LMO samples. Meanwhile, surface Mn dissolution causes structural damage and uneven Li diffusion, which in turn triggers partially destructive over-lithiation and Jahn–Teller distortion with Li_2_Mn_2_O_4_ generation. This state is confirmed with TEM results showing that Li_2_Mn_2_O_4_ predominately appears on the surface. Thereupon, LMO constantly suffers detrimental chemical cycles in tandem with irreversible phase transition behavior, severe Mn dissolution, and particle cracks, all of which constantly engulf spinel LiMn_2_O_4_ cathode capacity. Meanwhile, the increased Mn dissolution subsequently interferes with the anode SEI (Supplementary Fig. 20), which, in combination with cathode damage, shortens battery cycle life^18,53^. Despite unavoidable Mn dissolution LR-LMO observed relatively better cycling performances, due to a stabilized structure and decelerated Mn cathode dissolution. Absence of Mn_3_O_4_ in the charge process slows down Mn dissolution. Decelerated Mn dissolution and Mn(III) disproportionation effects subsequently prevent Li_2_Mn_2_O_4_ generation at discharge. These unprecedented discoveries not only clarify the interdependent relationship between phase transition and Mn dissolution but also emphasize the significance of structural stability on cathode cycle performance. Such insight provides theoretical guidance and practical routes for future cathode design.

### Full understanding of the LR-LMO structural advantage

**Fig. 6:**
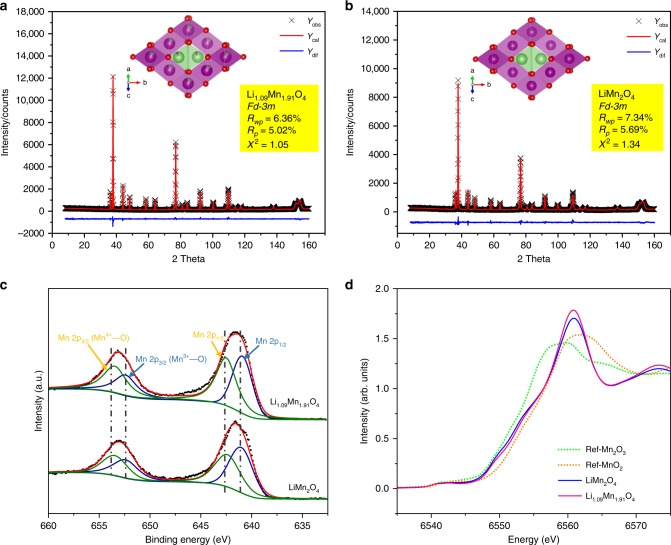
Accurate quantitative atomic occupancy analysis via neutron diffraction and the oxidation state analysis of Mn. **a**, **b** The neutron diffraction and Retvield refinement of LR-LMO and LMO powders. The inserted structure diagrams of LR-LMO and LMO. The green, purple, and red atoms represent Li, Mn, and O, respectively. **c** Mn 2p_1/2_ and 2p_3/2_ spectra and fitting results of LR-LMO and LMO samples. **d** Mn K edge XANES and fitting results of LR-LMO and LMO samples

Considering the greatly improved structural stability and electrochemical performance, of particular interest is to investigate why Li enrichment can enhance structural stability. To fully understand the advanced nature of LR-LMO, we further integrate Neutron diffraction and STEM to quantitatively monitor the structural discrepancy at the atomistic level of the two materials. Due to its good sensitivity to lithium, neutron diffraction was applied to precisely investigate the fine structures and atomic occupancies of LR-LMO and LMO. As shown in Fig. 6a, b and Supplementary Table 2, the neutron patterns of the two samples not only exhibit the characteristic Bragg peaks for a well-crystallized spinel structure with $Fd\bar 3m$symmetry but also some differences, carried out by Rietveld refinement using the GSAS package, in structural information detail such as the cell parameters and atomic occupancies. The refinement of LMO neutron diffraction (Supplementary Table 2) shows that Li and Mn respectively occupy site 8a and 16d at occupancies extremely close to 100%, which confirms the almost perfect spinel structure. In contrast, LR-LMO shows a smaller cell parameter and different occupancies, with 16.2% of lithium occupying the Mn site (16d) and 7.2% of Mn occupying the Li site (8a). A combination of the ICP and neutron diffraction refinement results conclude that the 7.2% of Mn found on the Li site of LMO was caused by an Li/Mn disorder. Similarly, the results also conclude that the 16.2% of Li found on the Mn site was caused by a 7.2% Li/Mn disorder as well as a 9.0% Li excess in the LR-LMO. Utilizing in situ XRD, LR-LMO with partial symmetry breaking is observed to behave in a manner similar to a solid-state reaction, which benefits a smaller volume change. This statement is confirmed with LMO and LR-LMO cell parameter change comparisons and precise investigation with in situ XRD. As shown in Supplementary Figs. 21 and 22, LMO with two distinct phases exhibits a huge cell parameter change, while LR-LMO following a solid-solution behavior shows a smooth cell parameter change. Based on the discussion above, we consider that the huge cell parameter change should be the potential external driving force of Mn(III) disproportionation, since it is found to occur at a high voltage. The impression of Mn(III) disproportionation of LR-LMO results from the solid-solution reaction and the small cell parameter during charge/discharge, benefiting the subsequent Mn dissolution and Li_2_Mn_2_O_4_ generation. Since Li saturation can be treated as Li doping, this statement is also extended to some doping elements such as Al, Mg, and Ti^53,54^. These doping elements partly break the symmetry of cathode structure and acting like structural pillars effectively relieve cell parameter changes, which is beneficial to the inhibition of irreversible phase transition and capacity fading.

**Fig. 7:**
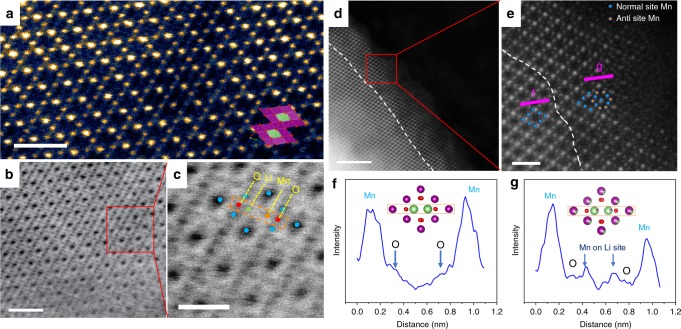
Visual atomistic-level observation of detailed structure and atomic occupancy for LR-LMO bulk and surface. **a** The cross-sectional STEM-HAADF imaging showing the atomic structure of LR-LMO bulk. **b** The cross-sectional STEM-ABF imaging of LR-LMO bulk. **c** Enlarged image of selected panel **b** areas. The green, blue, red, and orange atoms represent Li, Mn, O, and anti-site Mn, respectively. **d** The cross-sectional STEM-HAADF imaging showing the atomic structure of the LR-LMO surface. **e** Enlarged image of selected panel **d** areas. The blue and orange atoms represent normal Mn atom and anti-site Mn atom. **f**, **g** Corresponding atomic contrast curve of purple-bar f and purple-bar g labeled in panel **e**. Scale bars, 1 nm (**a**, **b**); 0.5 nm (**c**); 5 nm (**d**); and 1 nm (**e**)

Due to enormous atomic occupancy differences, an aberration-corrected STEM technique, with an annular bright-field (ABF) and high-angle annular dark-field (HAADF) detector, was employed to directly observe both the Li/Mn disorder distribution and crystallographic structure differences of the LR-LMO sample at the atomic scale. A typical scanning transmission electron microscopy with both annular bright-field (STEM ABF) imaging and high-angle angular dark-field (STEM HAADF) imaging can clearly capture the Mn sites; both the bright dots in the HAADF image and the black dots in the ABF image are indicative of Mn atom columns. Even though their contrasts are much weaker than those of Mn atom columns, oxygen atom columns can also be carefully observed in ABF imaging. Figure 7a viewed along the [110] direction shows the crystal structure of spinel LiMn_2_O_4_ exhibiting the columns of Mn atoms. Similarly, the ABF image found in Fig. 7b also shows a clear Mn atom column. Meanwhile, the column of O atoms can also be directly observed in the enlarged image (Fig. 7c). Interestingly, weak signals in the lithium site with contrasts quite similar to oxygen can be carefully observed in certain areas. It should be noted that the stacking density of oxygen is two times more than Li in spinel structures and that oxygen is heavier than Li. Therefore the Li site contrasts cannot be caused by Li, but are instead caused by Mn that comes from the Li/Mn disorder. This fact, along with atomic scale microscopy, directly verifies the existence of Li/Mn disorder in the bulk of LR-LMO.

LR-LMO surface atomic structures were further investigated via STEM-HAADF. Close examination of the surface regions revealed two types of local atomic-level structures on the LR-LMO sample, shown separated by dashed lines in Fig. 7d. Enlarged figures (Fig. 7e) show clear local structure information, with weak contrast present on Li sites. According to previous discussion in this paper, the weak contrast should also be attributed to Mn resulting from Li/Mn disorder. The amount of Li/Mn disorder on the surface is also significantly more than that of the bulk. Figure 7f, g shows the line profile, corresponding to the purple line f and g in Fig. 7e, which suggests heterogeneous Mn ion occupation on the tetrahedral sites due to the varying surface contrast. Therefore, compared to forming in the bulk, Li/Mn disorder prefers to form 3–5 nm layers distributed on the surface. Even with careful LMO sample observation (Supplementary Figs. 23 and 24), there is no trace of Li/Mn disorder on the particle surface or bulk.

The surface oxidation states of Mn were then examined by X-ray photoelectron spectroscopy (XPS) with a shallow detection scale (<3 nm). Figure 6c shows Mn 2p_1/2_ and 2p_3/2_ peaks of two samples at 640.9 and 653.1 eV, consistent with the data reported before^55,56^. Further fitting results showed that Mn^4+^ respectively occupied 47% and 59% of the LMO and LR-LMO sample surfaces (Fig. 6c), suggesting that LR-LMO surfaces with Li/Mn disorder and higher average Mn oxidation states form a stable LR-LMO reconstruction interface. This interface potentially blocks the direct contact of soluble manganese with electrolyte, which in turn inhibits manganese dissolution.

Finally, X-ray absorption spectroscopy was employed to probe the oxidation state and local bonding environment of Mn. Figure 6d shows the main edge of two samples in X-ray absorption near edge spectroscopy (XANES). After linear combination fitting, the detailed oxidation state of Mn revealed that Mn^4+^ respectively occupied around 56% and 51% in the LR-LMO and LMO samples which is consistent with the chemical component calculation results. Extended X-ray absorption fine structure (EXAFS) results reveal differences in Mn bonding length between LR-LMO and LMO. As shown in Supplementary Fig. 25, the Fourier transform for the first and second coordination shells of the two samples have two main peaks, corresponding to Mn–O and Mn–Mn bonding. We can see the LR-LMO sample exhibits smaller Mn–O bonding than LMO, suggesting that Li/Mn disorder shortens the distance of Mn–O bonding and enhances Mn–O stability. These results are in agreement with the cell parameters found through neutron diffraction refinement listed in Supplementary Table 1 and the suppression of Jahn–Teller distortion discussed above.

## Discussion

In summary, we jointly considered dynamic phase stability with TM dissolution in order to explore the detrimental roles of cathode material capacity fading. Based on in situ/ex situ XRD and Mn dissolution measurements, we revealed that stoichiometric LiMn_2_O_4_ suffers from a severe irreversible phase transition with the generation of unexpected phases and particle surface cracks, which greatly destroys surface stability and exacerbates Mn dissolution. Accelerated Mn dissolution, with the combination of Jahn–Teller distortion, in turn triggers irreversible phase transition, resulting in increased capacity fading. These two reactions form a detrimental chemical cycle that constantly engulfs the capacity of LMO. Li-rich LMO contains structural defects that effectively suppress these irreversible phase transformations by reducing cell parameters and inhibiting Jahn–Teller distortion during charge/discharge, thereupon achieving improved structural stability. In addition, Li-rich LiMn_2_O_4_ exhibits stable surface reconstruction that virtually inhibits Mn dissolution by blocking soluble manganese and electrolyte contact. These findings shed new light on the mechanistic properties of capacity fading in Mn-based materials and will serve as guidelines for new long life battery material designs.

## Methods

### Materials

Two samples of the spinel positive electrode material Li_1+*x*_Mn_2−*x*_O_4_ (*x* = 0 or *x* = 0.1) were prepared through solid-state reactions. Appropriate mole ratios of Mn_3_O_4_ and Li_2_CO_3_ precursor for LMO and LR-LMO were mixed through ball milling for 6 h. One percent molar excess of lithium was used due to the volatility of lithium at high temperatures. The mixtures were then heated for 20 h in air at 850 °C and naturally cooled. Afterwards, inductively coupled plasma-atomic emission spectrometry (ICP-AES) was used to measure the Li/Mn ratio for the original powders and the amount of Mn deposited on the Li metal after complete hydrochloric acid sample dissolution.

### Electrochemistry tests

For electrochemical testing, the active materials were mixed with carbon black and PVDF at 80:10:10 wt% ratios and ground in a mortar. In all, 2032 type coin cells were used to prepare lithium half cells. Celgard 2325 separators and 1.2 M LiPF_6_ in EC/EMC (3:7) electrolyte (GEN II) were used. The half cells were then cycled between 3.4 and 4.3 V vs. Li^+^/Li, using small powder amounts (∼10 mg) as cathode electrodes and lithium metal as an anode. Initial capacities for the two materials were tested with a C/10 rate, while cycle performances were tested with a C/5 rate after three cycles of activation. For the full cell, a 2032 coin cell was assembled using graphite as an anode and then cycled with a C/10 rate between 3.3 and 4.3 V. The N/P ratio for the full cell was around 1.2 and the commercial graphite was provided by Shenzhen BTR New Energy Materials Inc.

### Neutron diffraction tests

Neutron diffraction experiments were initially performed using the BT-1 high-resolution powder diffractometer at the NIST Center for Neutron Research. Powder samples were put in cylindrical v.anadium cans and measured in transmission geometry at *λ* = 1.5944 Å (calibrated using Na_2_Ca_3_Al_2_F_14_ as a reference). Rietveld refinement of ex situ neutron diffractions were performed with *GSAS* software packages.

### Synchrotron XRD, XAS, and XRF measurements

In situ, time-resolved, high-energy synchrotron XRD (HEXRD) measurements during cycling were performed at beamline 11-ID-C of the Advanced Photon Source (APS) at Argonne National Laboratory. Exhibiting high penetration and low absorption, synchrotron HEXRD precisely reflects bulk sample structure properties in real time and realistic conditions. This promotes observation of tiny phase changes that are unobservable with lab scale XRD because of poor background noise and time resolution limitations. A high-energy X-ray with a beam size of 0.2 mm × 0.2 mm and wavelength of 0.1173 Å was used to obtain two-dimensional (2D) diffraction patterns in the transmission geometry. All the patterns of HEXRD were plotted with a 2-theta range of 0–10. X-ray patterns were recorded with a Perkin-Elmer large-area detector placed at 1800 mm from the battery cells. The 2032-coin cells exhibited a 3 mm hole suitable for X-rays to pass through and diffraction patterns were collected every 10 min. For ex situ XRD sample preparation, we scraped powders from the cycled electrodes and sealed the samples with Kapton tape to avoid air exposure. The Rietveld refinements for ex situ XRD were performed with TOPAS software packages. The XANES for Mn K edge was performed at the APS on the bending-magnet beamline 9-BM-B. The X-ray photon energy was monochromatized by an Si(111) double-crystal monochromator. Higher-order harmonic contaminations were eliminated by detuning the monochromator to reduce the incident X-ray intensity by approximately 30%. All spectra were collected at room temperature in the transmission mode. Ex situ XRF measurements were performed at the 2-ID-E beamline of the APS at Argonne National Laboratory. The sample was excited by 10 keV X-ray photons with a submicron beam size. Emitted XRF signals were then detected by an energy-dispersive detector while the sample was raster scanned. The minimum detection limits of synchrotron-based X-ray fluorescence are much higher than ppm levels sufficient to detect TM elements such as Mn, Ni, and Fe. The 2D element concentrations were then calculated by MAPS^57^. A region of graphite anodes with a size of 500 µm × 500 µm was randomly selected for mapping.

### X-ray photoelectron spectroscopy measurement

The X-ray photoelectron spectra (XPS) for various samples were collected with an ESCA Lab 220I-XL XPS system equipped with an ion etching system, domain XPS, and ion diffraction analysis system. This system used a focused, monochromatic Al Kα X-ray (1486.7 eV) source for excitation and as a spherical section analyzer. All of the spectra were charge referenced using the C 1s line at 284.6 eV for comparison.

### Transmission electron microscopy measurement

The high-resolution transmission electron microscopy images of LR-LMO and LMO were obtained from a FEI Titan 80-300ST (with a spherical and chromatic aberration imaging corrector and a JEM-3200FS, JEOL), an aberration-corrected STEM (JEOL ARM 200CF) and an Argonne Chromatic Aberration-corrected TEM (ACAT) (an FEI Titan 80-300ST with an image aberration corrector to compensate for both spherical and chromatic aberrations) at an accelerating voltage of 200 kV.

## Supplementary information

Supplementary Information

## Data Availability

The data that support the findings of this study are available from the corresponding authors upon reasonable request.
