# Supplementary material for: Correlation between manganese dissolution and dynamic phase stability in spinel-based lithium-ion battery
Source: Nat Commun. 2019 Oct 17;10:4721. doi: 10.1038/s41467-019-12626-3 (PMC6797712; doi:10.1038/s41467-019-12626-3)
Supplement: Supplementary file 1 — Supplementary Information [file 41467_2019_12626_MOESM1_ESM.pdf]

Supplementary information

Correlation between Manganese Dissolution and Dynamic Phase Stability in Spinel-based Li-Ion Battery

Liu et al.

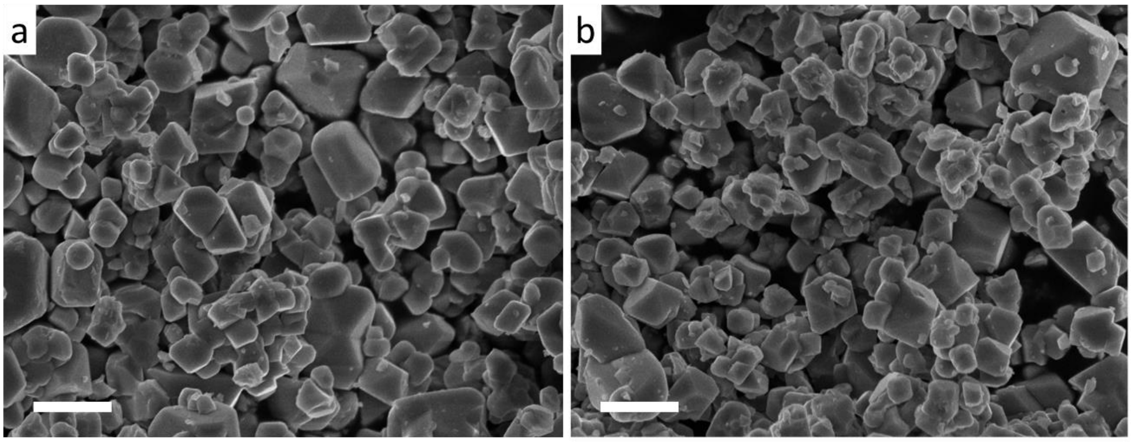

Supplementary Figure 1 | The morphology measurements of LMO and LR-LMO samples. a and **b**, The SEM imaging of LR-LMO and LMO. These two samples exhibit particle sizes and morphology. Scale bars, 1 μm (**a**, **b**).

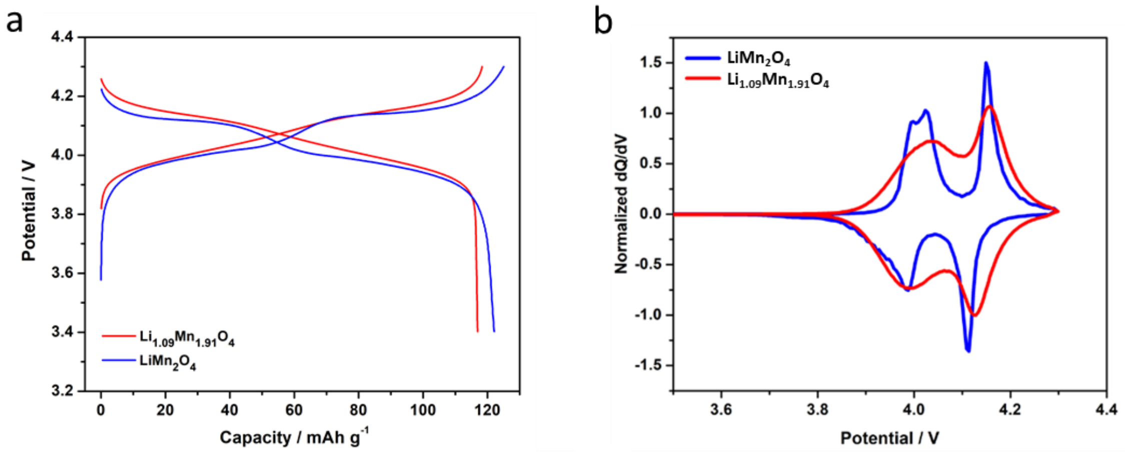

Supplementary Figure 2 | Electrochemical measurements in half cells. a, The charge and discharge curve of LMO and LR-LMO with voltage range of 3.4V-4.3V at 0.1C rate current. **b,** The dQ/dV curve of LMO and LR-LMO at 0.1C rate current.

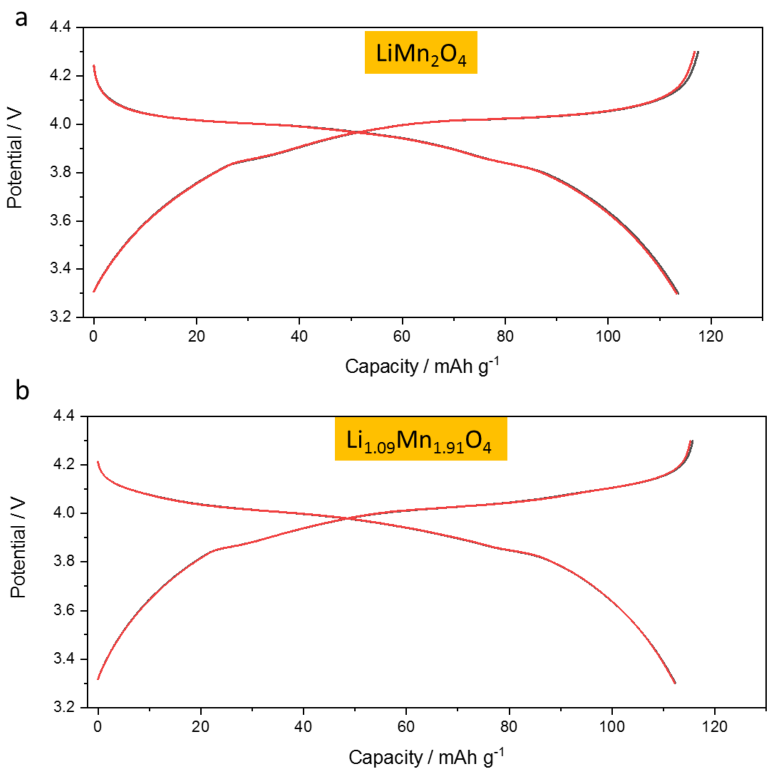

Supplementary Figure 3 | Electrochemical measurements in full cells. a and **b**, The charge and discharge curve of LMO and LR-LMO full cell with voltage range of 3.3V-4.3V at 0.1C rate current.

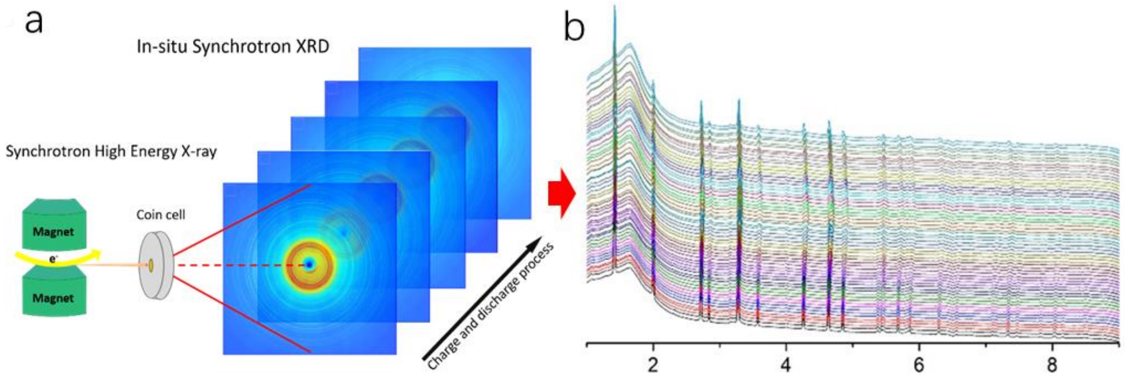

Supplementary Figure 4 | The schematic of in-situ synchrotron HE-XRD. a, The schematic of *in-situ* synchrotron HE-XRD and the home-made coin cell with a 3mm hole. **b**, The in-situ XRD curve of the first charge and discharge process for LR-LMO.

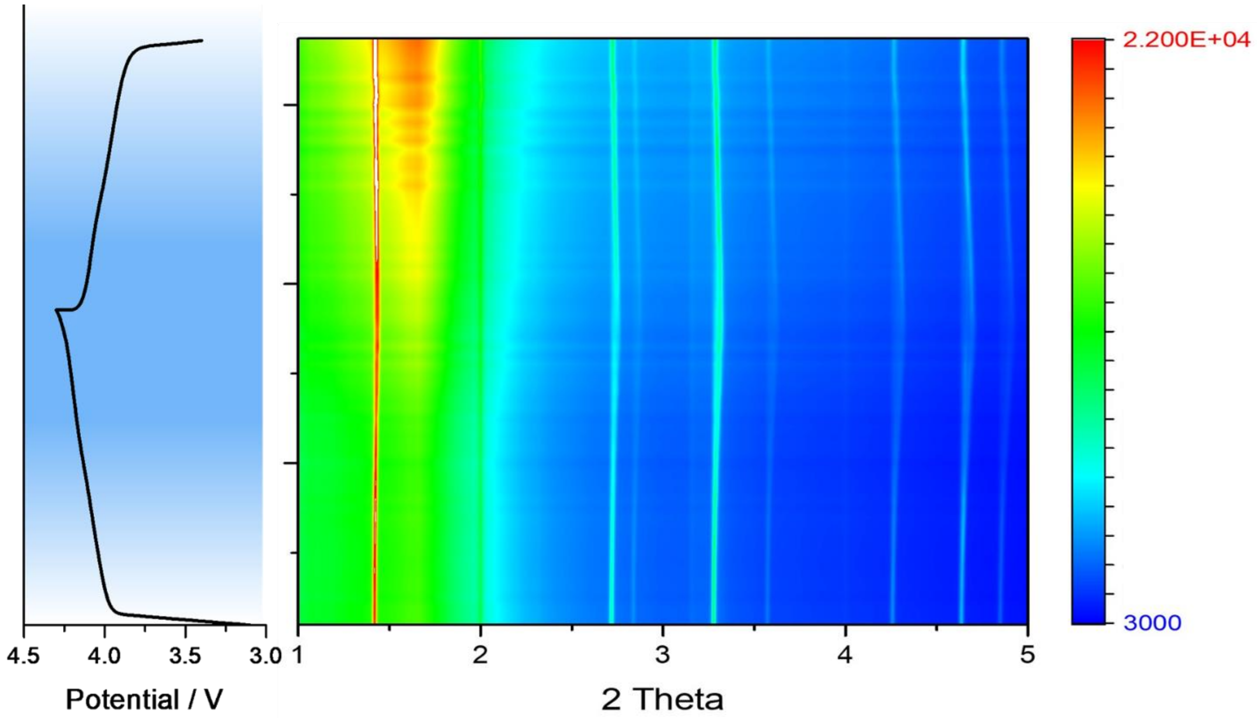

Supplementary Figure 5 | Contour plots of *in-situ* XRD of the first charge/discharge and the corresponding voltage profile of LMO sample. The LR-LMO exhibits a solid-solution type insertion/deinsertion behavior during charge and discharge. Most of peaks smoothly shift to right upon charge and shift to left upon discharge because the valance of Mn increases with Li deinsertion, but decreases with Li insertion.

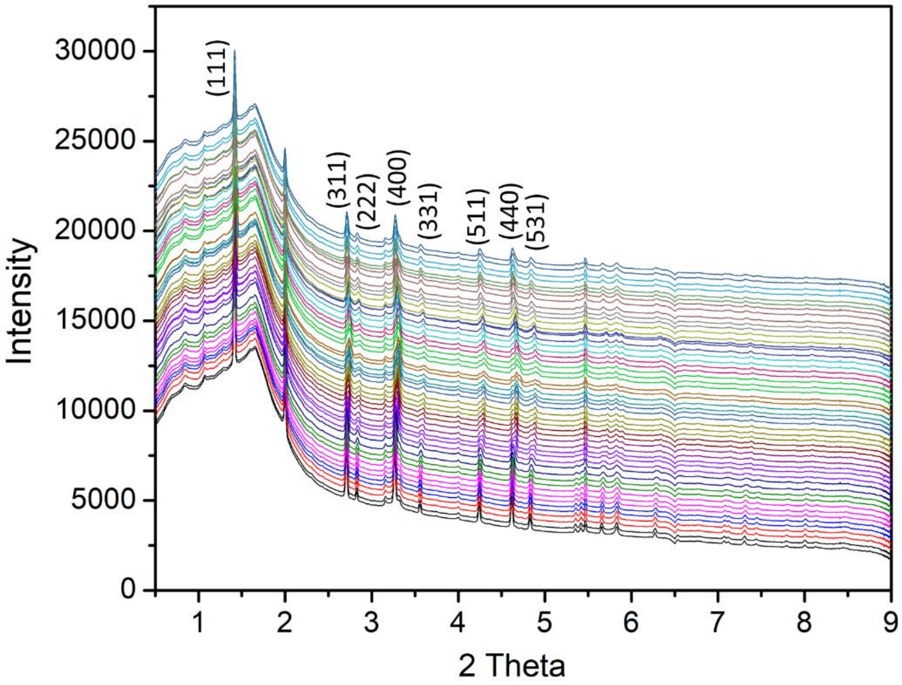

Supplementary Figure 6 | The *in-situ* XRD patterns of the first charge/discharge for LMO. The obvious phase transitions can be observed from *in-situ* XRD pattern, particularly in the 2-theta range of 3.0-5.0.

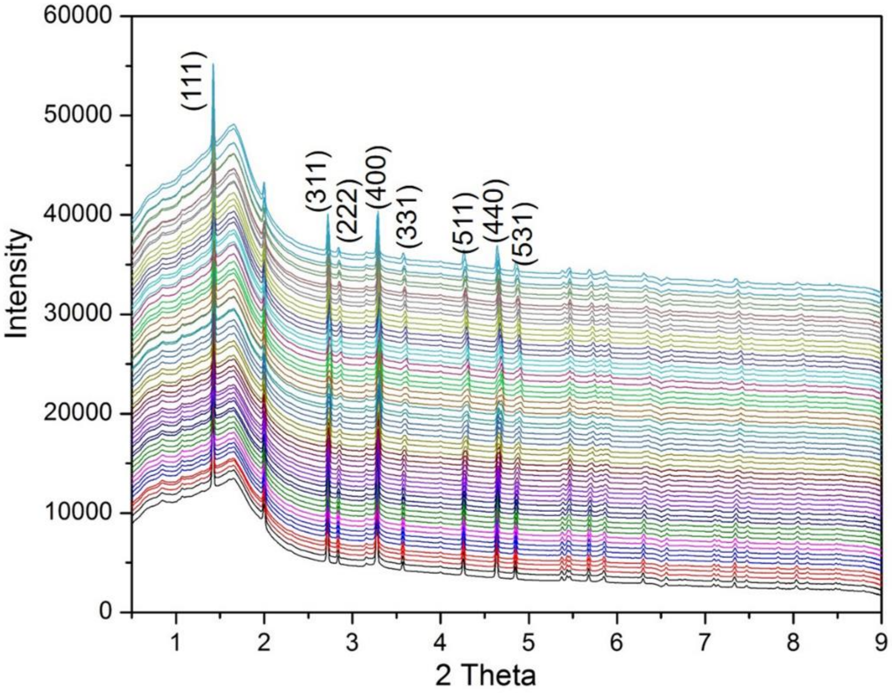

Supplementary Figure 7 | The *in-situ* XRD patterns of the first charge/discharge for LR-LMO. The LR-LMO exhibits a solid-solution type insertion/deinsertion behavior during charge and discharge, and no phase transition was visible in the in-situ XRD pattern.

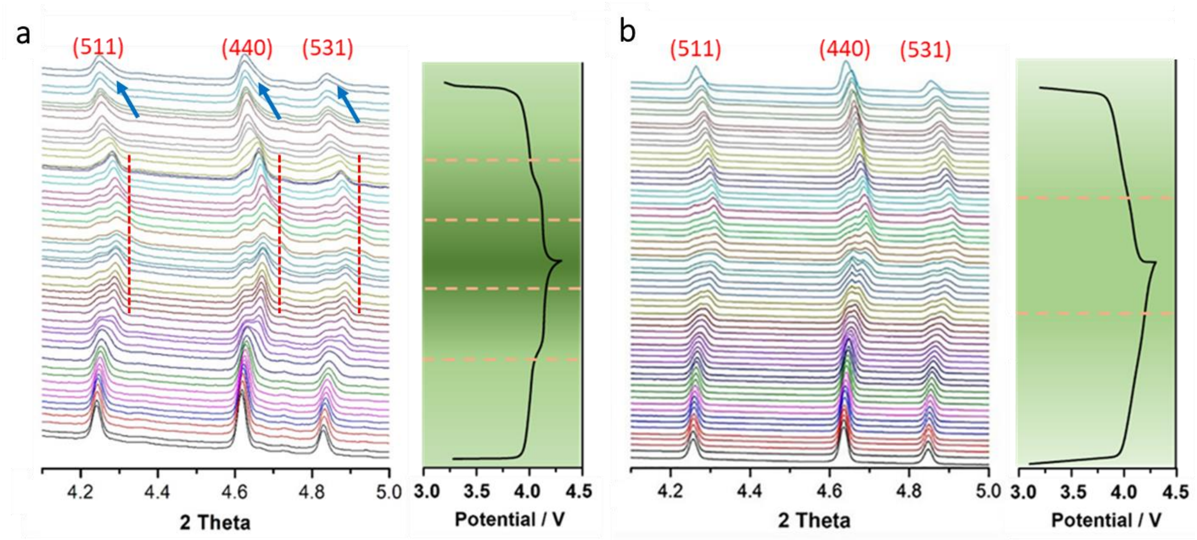

Supplementary Figure 8 | The enlarged XRD patterns of Bragg peaks (511), (440) and (531). a, The enlarged figure of Bragg peaks (511), (440), and (531) and the corresponding voltage profile for LMO. **b,** The enlarged figure of Bragg peaks (511), (440), and (531) and the corresponding voltage profile for LR-LMO.

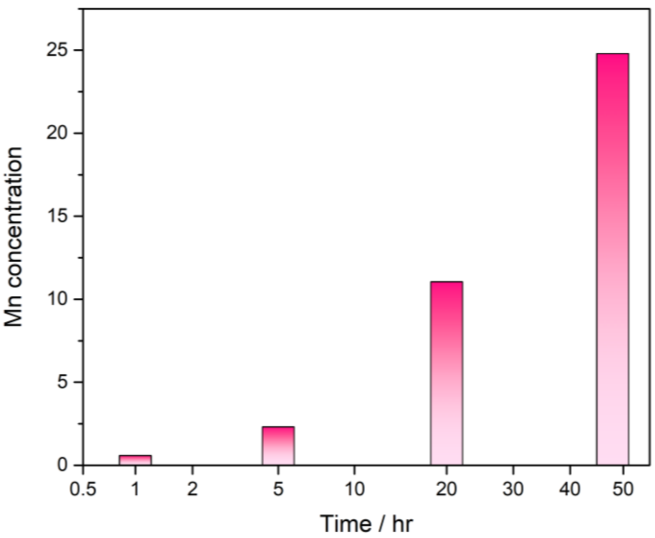

Supplementary Figure 9 | Mn_3O_4 chemical dissolution measurement in organic electrolyte (1.2 M $LiPF_6$ in EC / EMC (3:7) electrolyte).

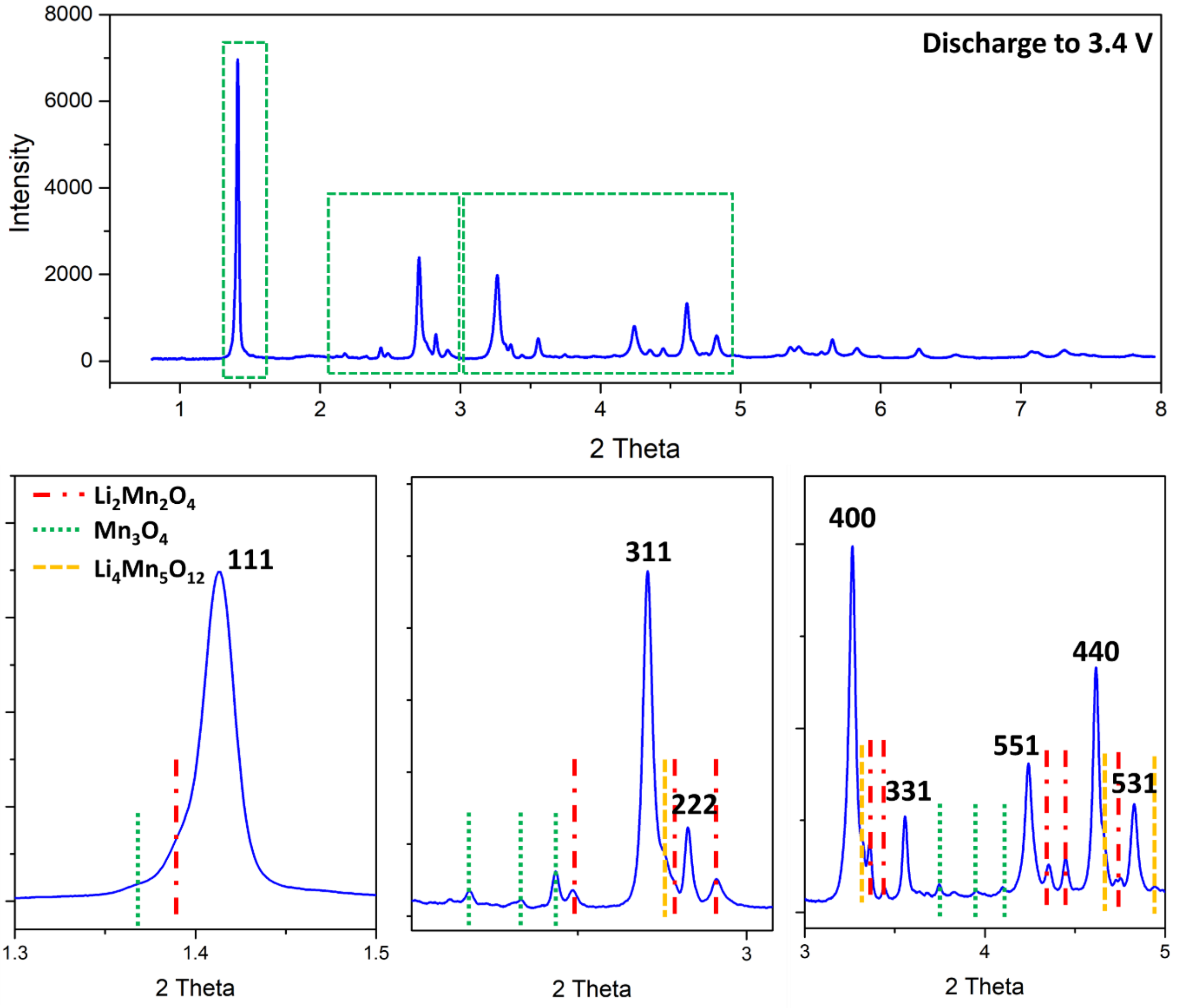

Supplementary Figure 10 | The *ex-situ* XRD for LMO after first discharge, and the enlarged figures with qualitative phase analysis.

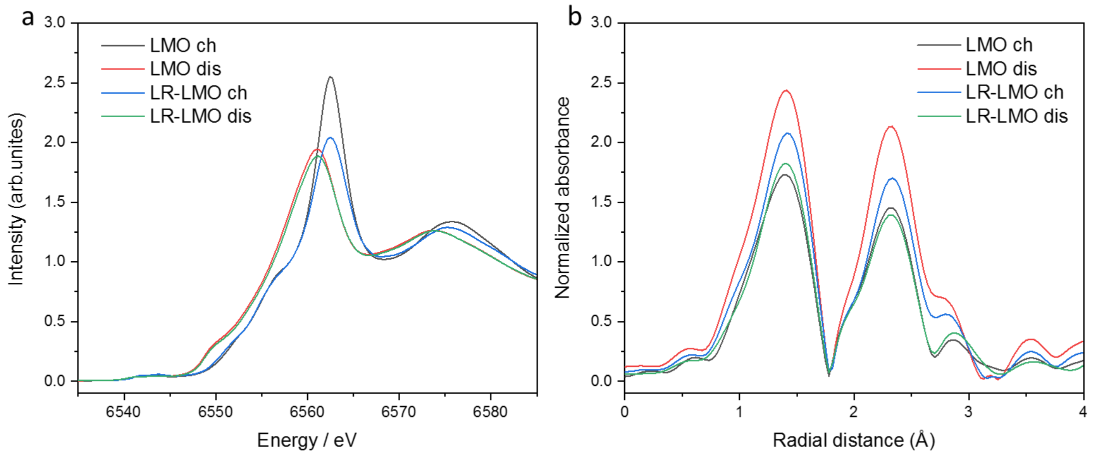

Supplementary Figure 11. The ex-situ XAS measurements at different states of charge. a. Mn K-edge XANES for LR-LMO and LMO samples at different potentials. b. Mn K-edge EXAFS for LR-LMO and LMO samples at different potentials. The main edge of both LMO and LR-LMO shifted to higher energy upon charge, and shifted to lower energy upon discharge, corresponding to Mn valance increasing and decreasing respectively (Supplementary Fig.11a). It is worth noting that even though the overall Mn valance changes of two samples are extremely consistent during charge and discharge, the main edge of LMO upon discharge visually shifts to lower energy in comparison with LR-LMO. This phenomenon should correlate to the average Mn valance decrease caused by a small amount of phase transformation from $LiMn_2O_4$ to $Li_2Mn_2O_4$ under the influence of *Jahn-Teller* distortion. We also conducted EXAFS to compare the fine structure of two samples at different state of charges. Supplementary Fig. 11b shows that the Fourier transform magnitudes for the first shell coordination of the two samples have two main peaks, corresponding to Mn-O and Mn-Mn bonding. There is no substantial shift in the Mn *K*-edge EXAFS spectra during charge and discharge. However, the peak intensity of the FT of the Mn *K*-edge EXAFS spectrum in LMO sample shows a large increase at charge. The peak amplitude of the FT is mainly related to the valance, the coordination number and local atomic density around the target atom[1,2]. The large increase of the FT peak intensity may partly result from a possible local atomic density increase due to the phase transition from $LiMn_2O_4$ to Mn_3O_4 and $Li_4Mn_5O_{12}$.

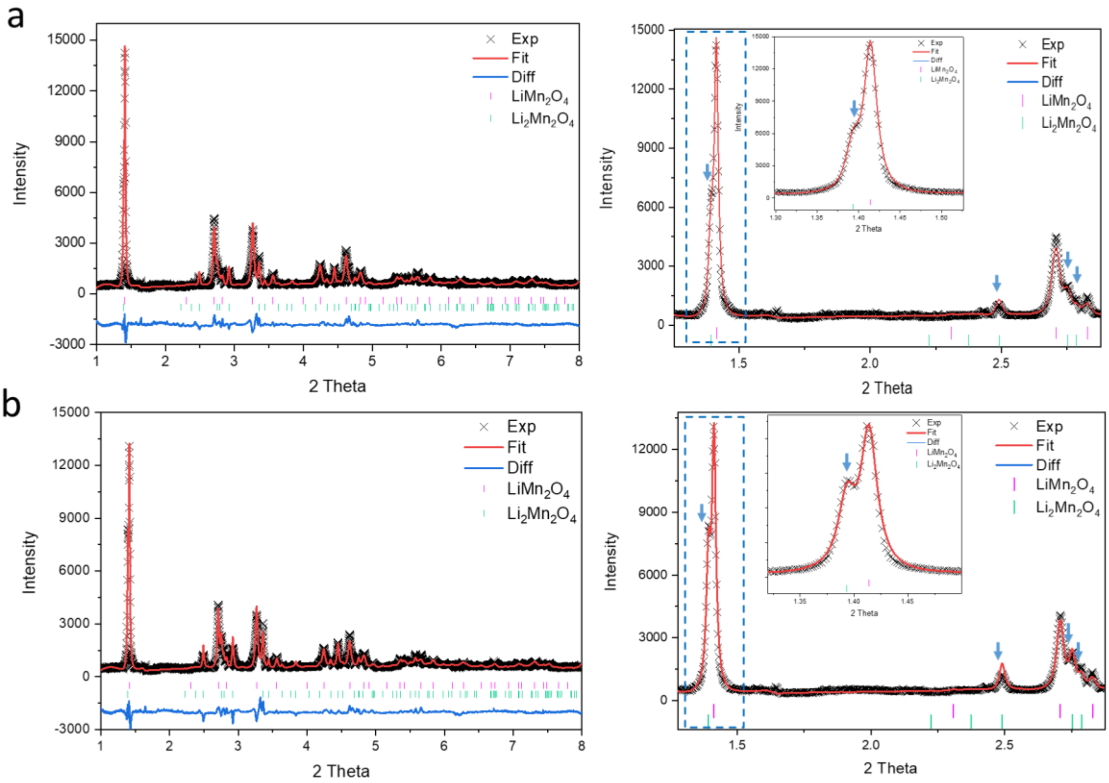

Supplementary Figure 12 | The XRD refinements of cycled electrodes. a and **b,** The refinement for XRD pattern of LMO after 25 and 50 cycles. Phase I and Phase II represent spinel $LiMn_2O_4$ (*Fd-3m*) and $Li_2Mn_2O_4$ ($I4_1$/amd), respectively. To further improve the XRD quality and the refinement accuracy, we collected powders from a total of 10 electrodes, and quantified the structural transformation with two samples of different cycles. As shown in Supplementary Fig. 12, some additional peaks are both present on the two XRD curves that exactly watch with $Li_2Mn_2O_4$. The refinement results with better accuracy confirms that 22% and 35% $Li_2Mn_2O_4$ were respectively quantified on the 25 cycles and 50 cycles electrode.

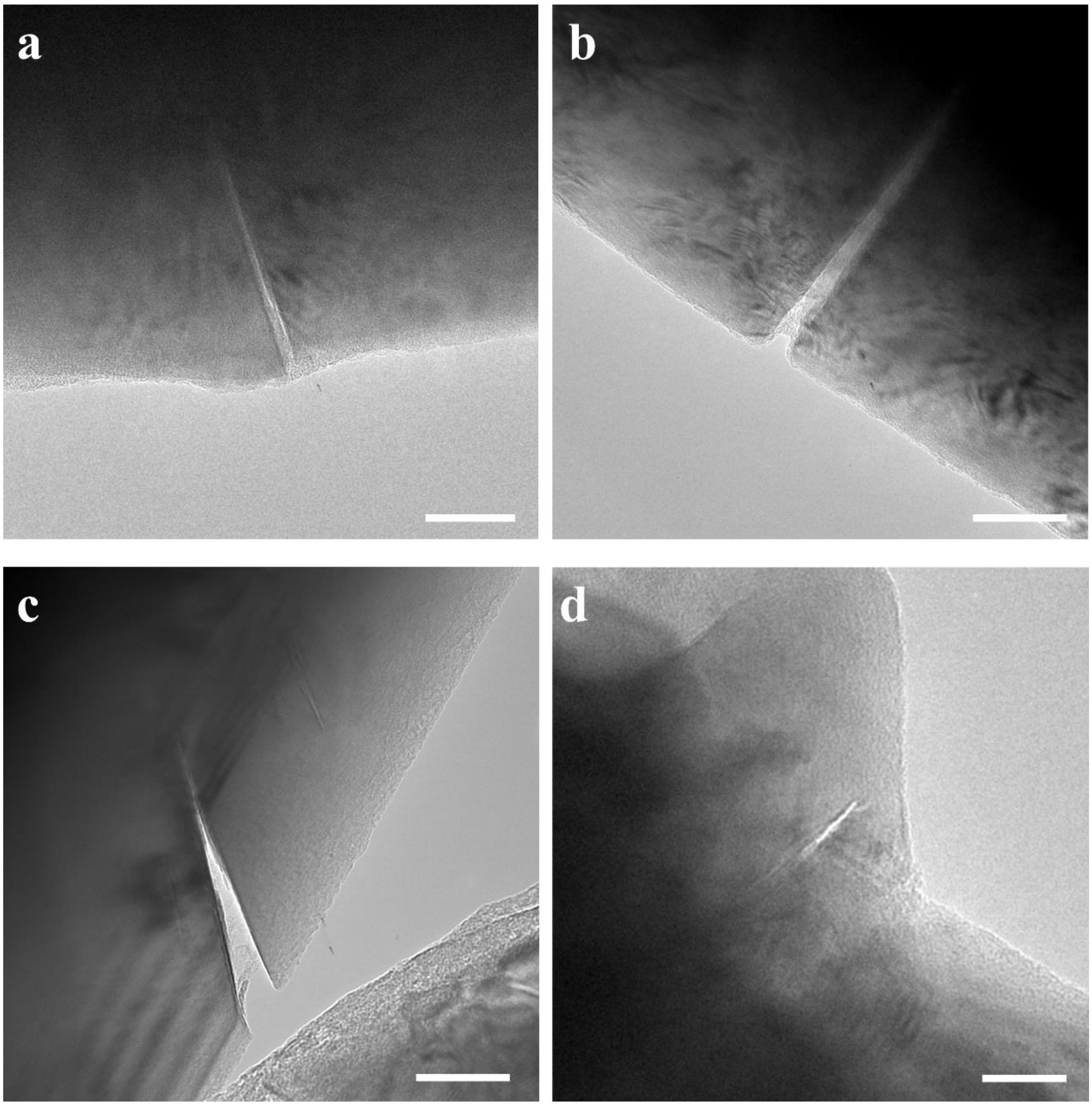

Supplementary Figure 13 | The observation of particle cracks with TEM imaging. (a), (b), (c) and (d) Low-magnification TEM images of cracks captured in different particles. Scale bars, 30 nm (**a**); 40 nm (**b**); 50 nm (**c**); and 20 nm (**d**). For TEM sample preparation, we scraped the powders from the cycled electrodes without grinding, then ultrasonically dispersed it with a small amount of alcohol. As shown in Supplementary Fig.13, we carefully conducted the TEM measurements on several particles scraped from cycled electrodes. Similar cracks appear constantly on the surface of the particles. More interestingly, we observed that the crack also generated inside the particles as shown in Supplementary Fig.13d, which strongly supported that the particle cracks were assuredly caused by the phase transformation and subsequent large change in cell parameter.

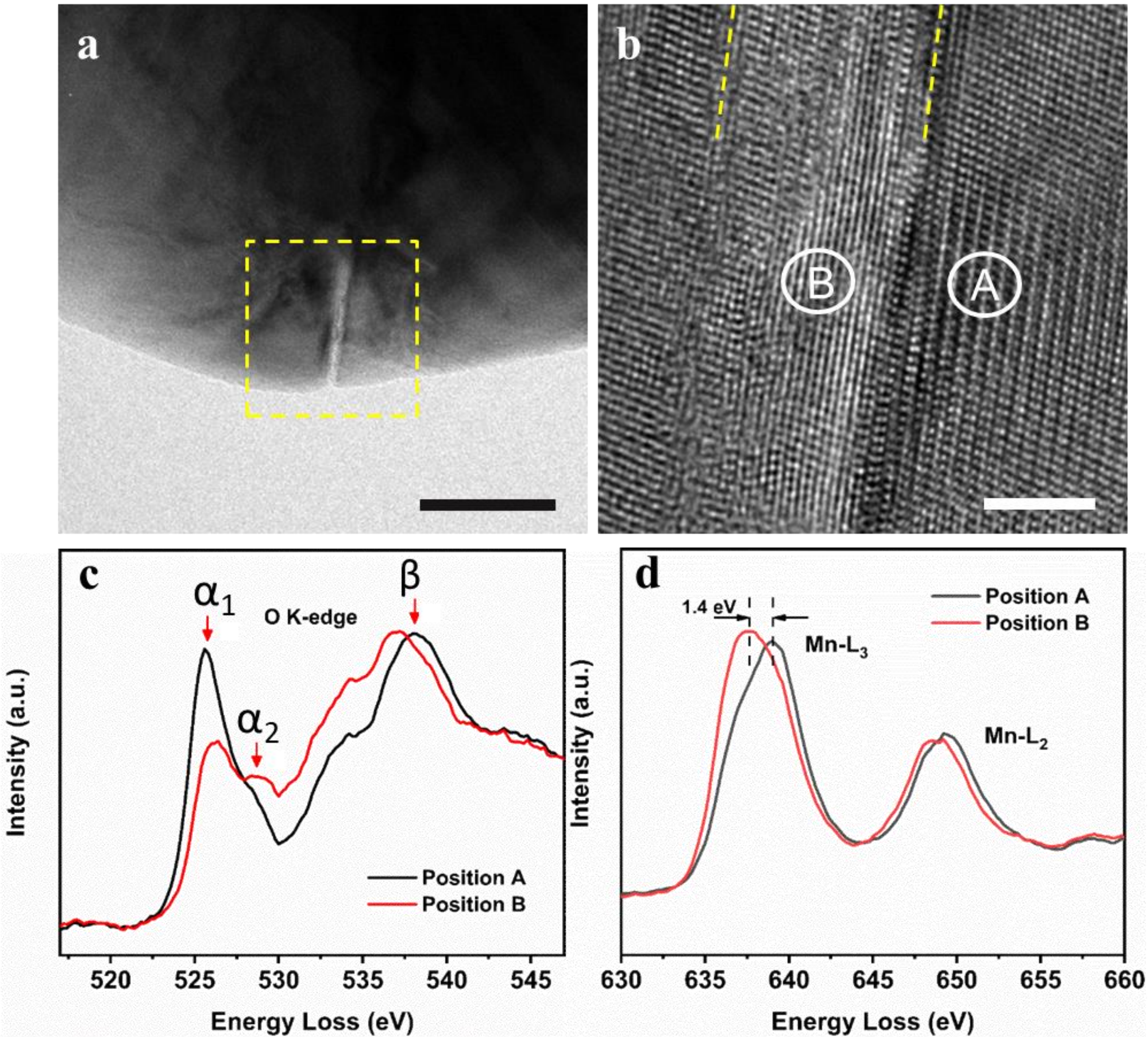

Supplementary Figure 14 | The TEM imaging and EELS measurements. a and **b,** Low-magnification TEM of cycled particle and corresponding HRTEM (b) images at a crack in $LiMn_2O_4$ after 25 cycles. **c** and **d,** Typical EELS spectra of O K-edge and Mn L-edges measured at bulk (position A) and crack (position B) as shown in **b**. Scale bars, 50 nm (**a**); 3 nm (**b**). We also conducted EELS spectra measurements to investigate the structural evolution and valence change of spinel particle surface. A low-magnification TEM image in Supplementary Fig. 14a shows a typical crack appearing on a $LiMn_2O_4$ particle after 25 cycles. HRTEM image of the crack shows an obvious difference in lattice fringes. More importantly, the EELS spectra of O *K*-edge (c) and Mn *L*-edge exhibits a dramatic change at position A (bulk) and position B (crack), indicating Mn valance change from position A (bulk) to position B (crack). Supplementary Fig. 14d shows that both of the peaks Mn-L_3 and Mn-L_2 exhibit chemical shifts towards lower energy loss and a higher intensity ratio of L_3/L_2, which directly demonstrates the decrease of Mn valence at the crack.[3,4] From the O-*K* edge spectra shown in Supplementary Fig. 14c, we can observe two main peaks labeled as α and β. Compared to Position A, the intensity of peak α_1 decreases and α_2 increases, and peak α_1 shifts towards higher energy loss and peak β shifts towards lower energy loss at the Position B. As a result, the EELS O-*K* edge exactly matches with that of Mn_3O_4 reported in the earlier literature. Thus, $Li_2Mn_2O_4$ and Mn_3O_4 are both certified appearance on the side of cracks in cycled particle of $LiMn_2O_4$.

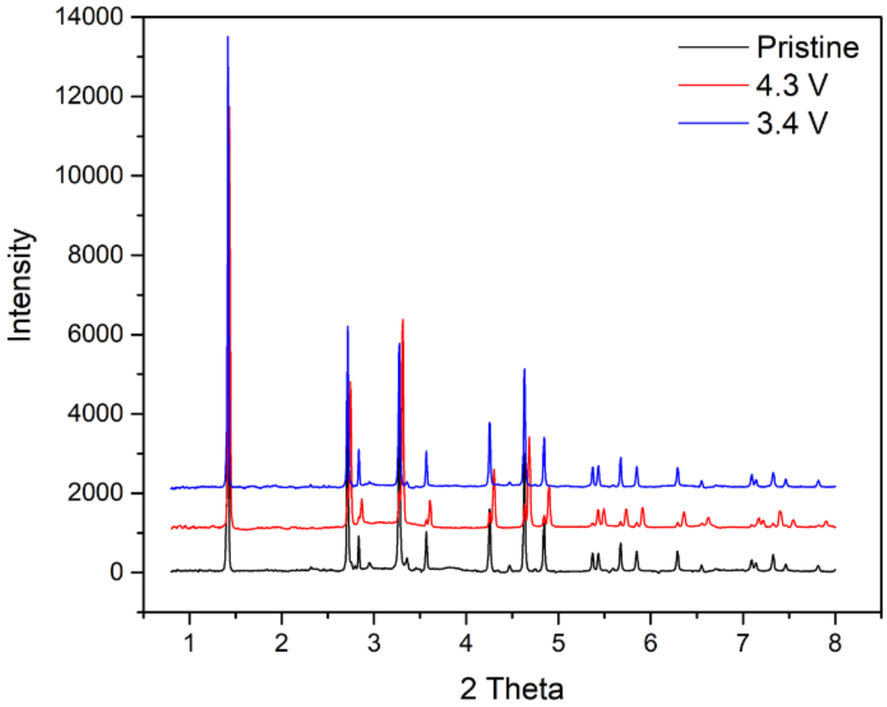

Supplementary Figure 15 | The *ex-situ* XRD of LR-LMO at different potentials of the first cycle.

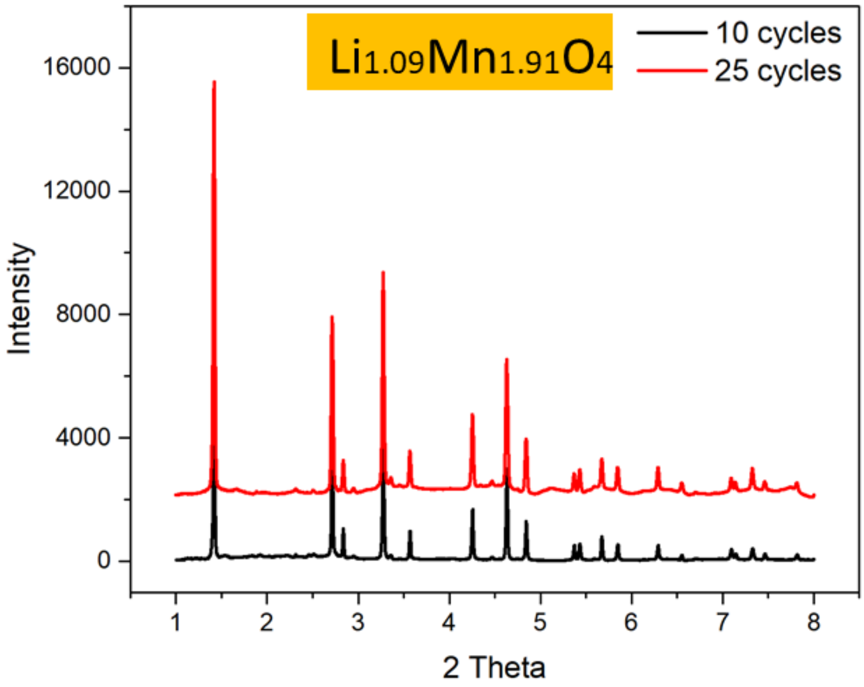

Supplementary Figure 16 | The *ex-situ* XRD of LR-LMO at different cycles.

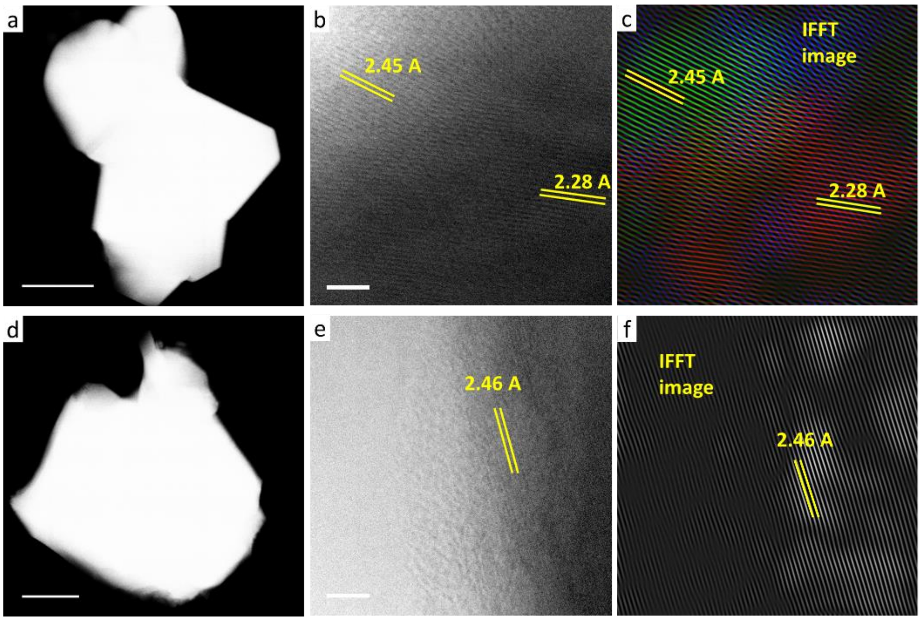

Supplementary Figure 17 | The TEM imaging of two LR-LMO particles after 25 cycles; **a** and **d,** Low-magnification TEM of cycled particle; **b** and **e,** the high resolution TEM observations of two particles surfaces; **c** and **f,** the corresponding IFFT images with clear lattices. Scale bars, 0.5 µm (**a**, **d**); 2 nm (**b**, **e**).

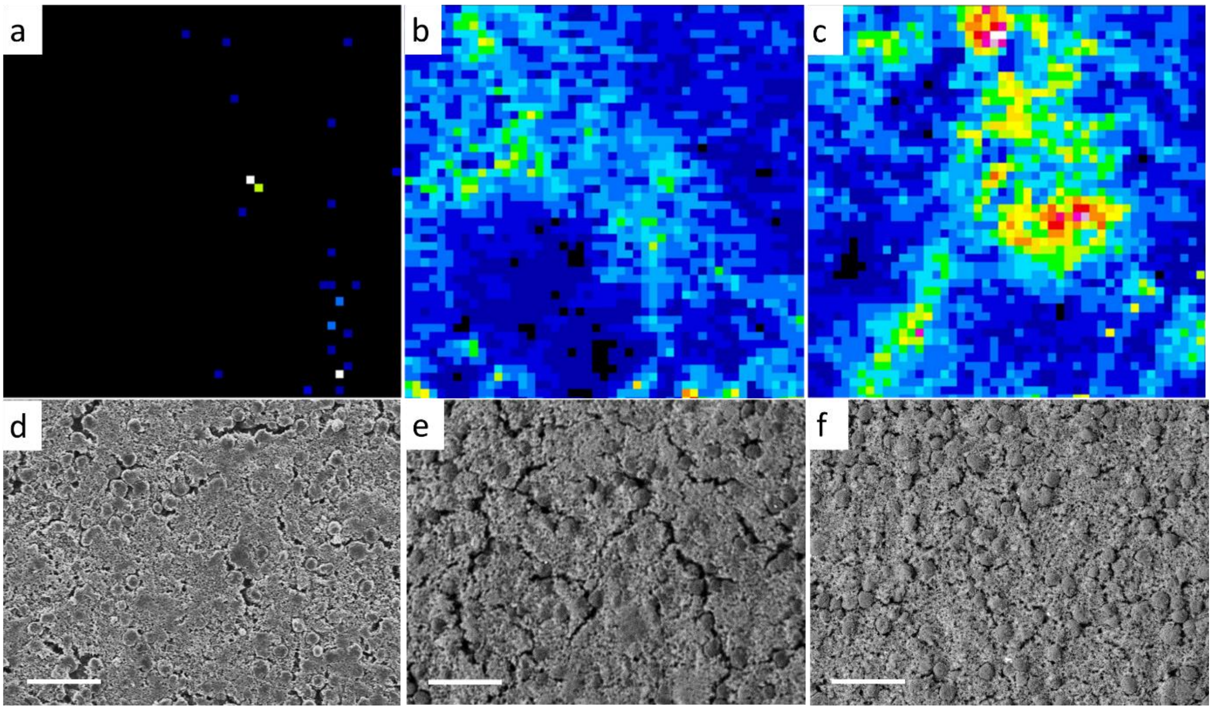

Supplementary Figure 18 | XRF two-dimensional Mn Kα elemental maps for graphite electrode disassembled from spinel $LiMn_2O_4$ / graphite full cell and their corresponding SEM images. a, The graphite electrode before cycling. **b**, The graphite electrode of LR-LMO / graphite full cell after 50 cycles. **c**, The graphite electrode of LMO / graphite full cell after 50 cycles. Two-dimensional Mn *Kα* elemental maps for graphite electrode. **d, e** and **f** are their corresponding SEM images. Scale bars, 100 μm (**a**, **b**, and **c**).

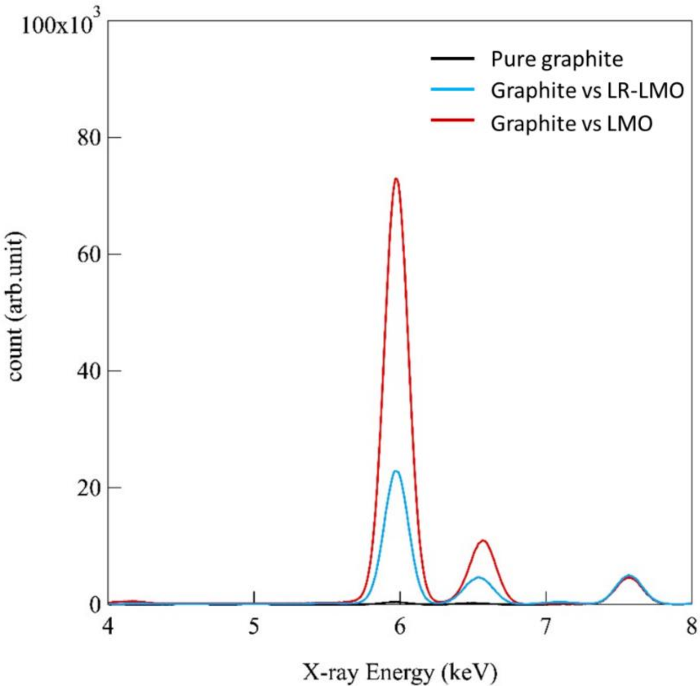

Supplementary Figure 19 | The spectra of XRF two-dimensional Mn *Kα* elemental maps for graphite electrode of the LMO / graphite full cell. We employed synchrotron-based X-ray fluorescence (2 ID-E, APS, Argonne National Laboratory) to qualitatively compare the Mn deposition content on anode of two samples. A region of graphite anodes with a size of 500 µm × 500 µm was randomly selected for mapping.

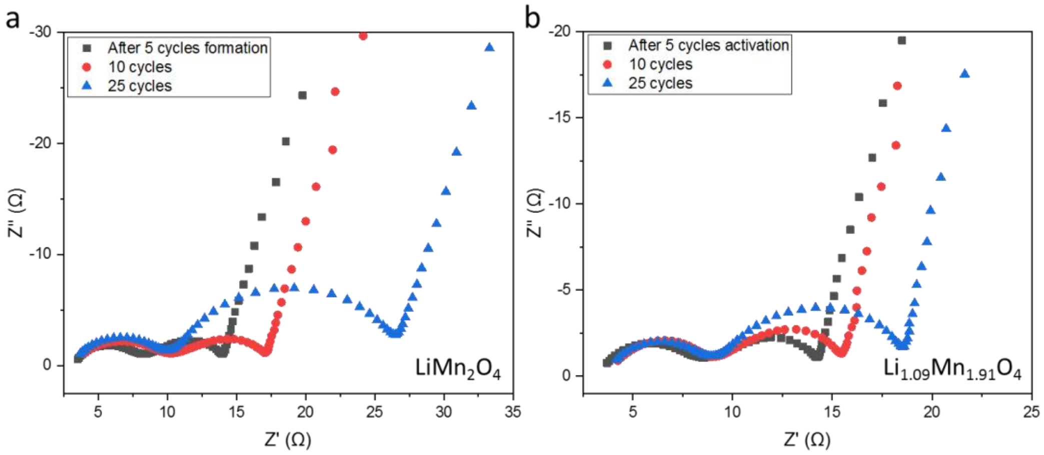

Supplementary Figure 20 **| The AC impedance results. a** and **b**, Nyquist plots of full cell as a function of cycle numbers for LMO and LR-LMO. We provided the AC impedance data to compare the SEI formation for these two cathode materials here. As shown in Supplementary Fig. 20, the impedance of the LMO||graphite full cell typically includes two semicircles in the high-frequency region and a line in the low frequency region, which corresponds to two serially connected parallel R_{ct} elements and the Warburg element, respectively.[5] The first semicircle (in higher-frequency region, as shown in the insertion of Supplementary Fig. 20a) remains almost the same from the initial to 25th cycle, whereas the second one (at lower-frequency region) constantly increases with cycle number. As R_{ct} represents the Li-intercalation reaction impedance, which is mainly determined by intrinsic properties of the active material, electrode SOC and temperature, it is reasonable to assume that R_{ct} tested here remains constant with cycling. On the other hand, the SEI layer will get thicker with the cycling of the cell, thereby changing R_{SEI}. We, therefore, assigned the first semicircle to the R_{ct} element and the second one to the R_{SEI} element.[6] As a result, the R_{SEI} of LMO full cell increases notably with cycling, whereas that of LR-LMO full cell just slightly raised. This result clearly indicates that more Mn ions dissolve from LMO sample, and damage the SEI formed on the graphite, leading to the dramatic increase of full cell impedance.

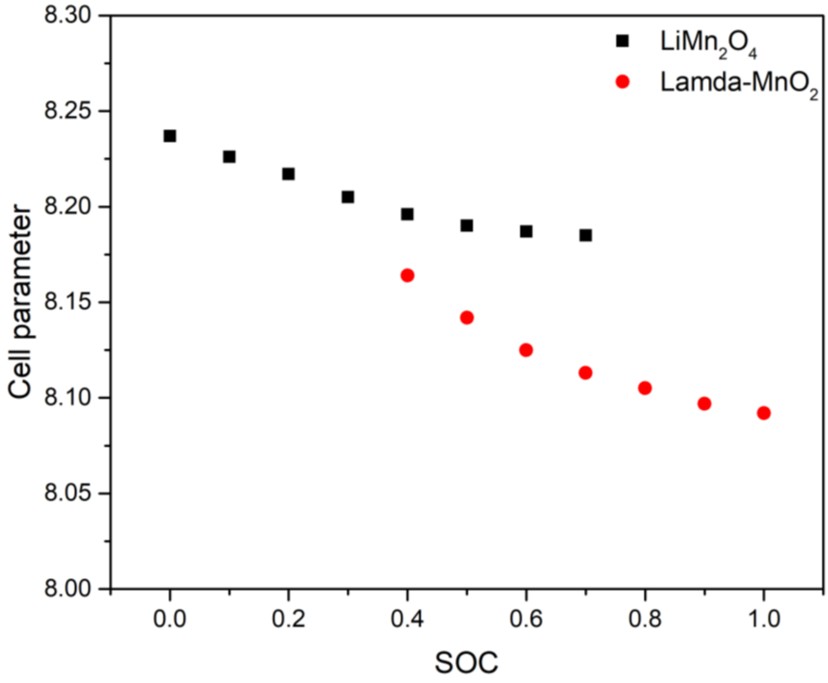

Supplementary Figure 21 | The cell parameter changes of LMO during the first charge process.

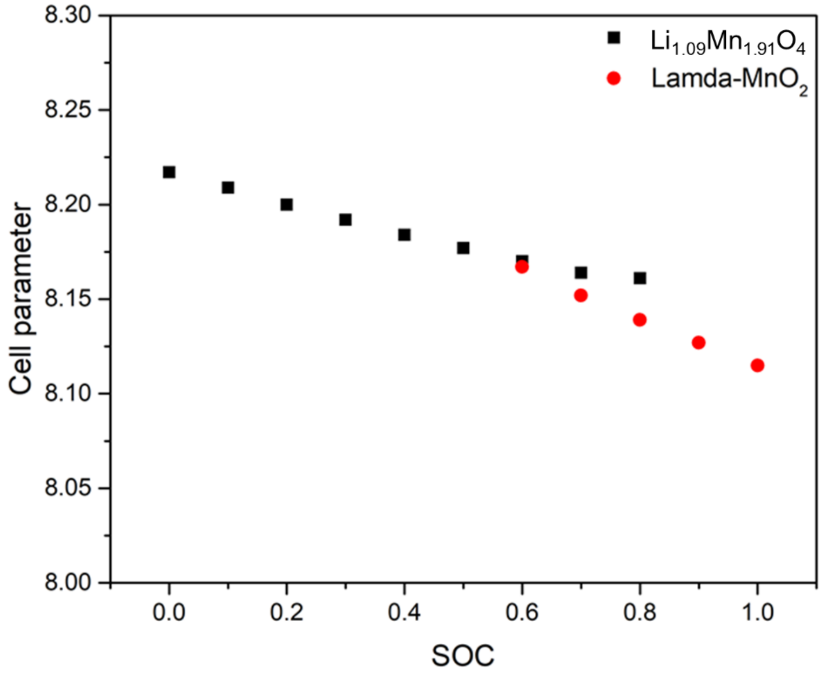

Supplementary Figure 22 | The cell parameter changes of LR-LMO during the first charge process.

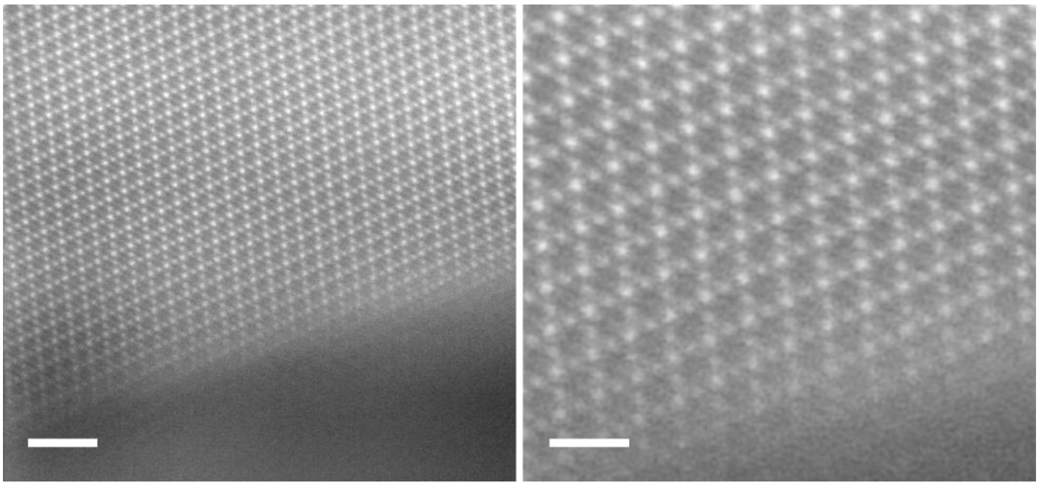

Supplementary Figure 23 | Cross-sectional HAADF-STEM imaging showing the atomic structure of the LMO surface. Scale bars, 2 nm (**a**); 1 nm (**b**).

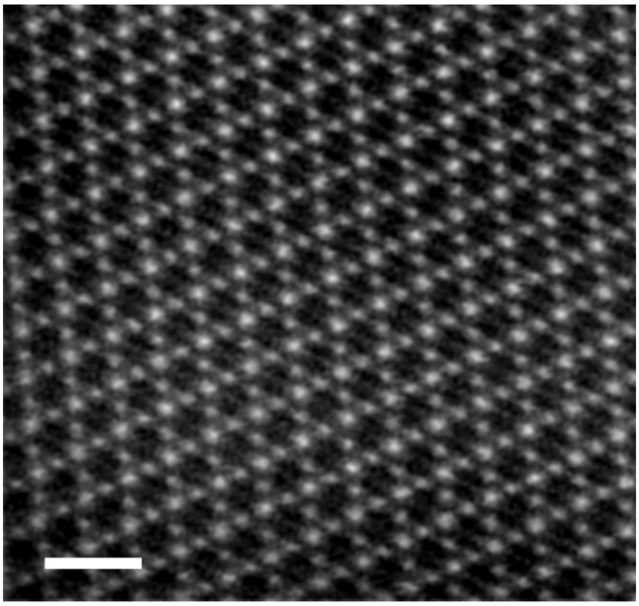

Supplementary Figure 24 | Cross-sectional HAADF-STEM image showing the atomic structure of the LMO bulk. Scale bars, 1 nm.

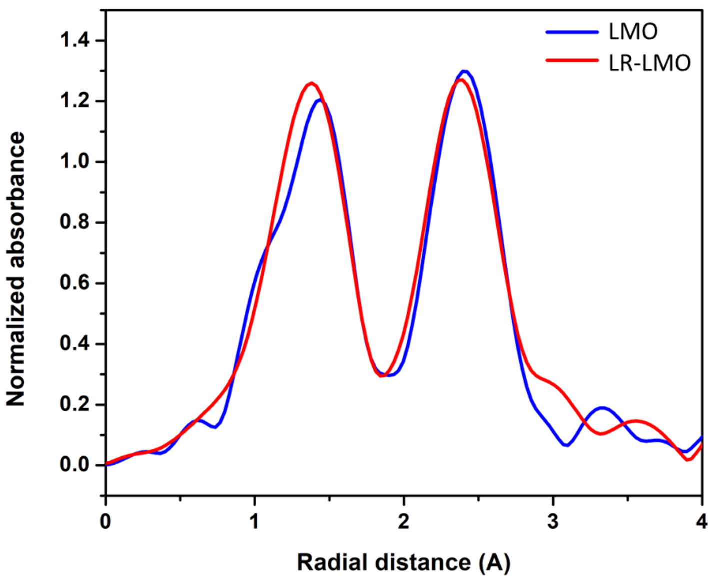

Supplementary Figure 25 | Mn *K*-edge extended x-ray absorption fine structure (EXAFS) of LR-LMO and LMO samples.

Supplementary Table 1 | The inductively coupled plasma-atomic emission spectrometry results of original LR-LMO and LMO.

Samples	The molar ratio of Li/Mn	Molecular formula
LR-LMO	0.571	$Li_{1.09}Mn_{1.91}O_4$
LMO	0.504	$LiMn_2O_4$

Supplementary Table 2 | The refinement results of the neutron diffraction of LR-LMO and LMO.

Sample	Element	Site	Occupancy	Biso
LR-LMO	Li1	8a	0.927	1.841
	Mn1	16d	0.919	0.719
	O1	32e	1.000	1.285
	Li2	16d	0.081	0.719
	Mn2	8a	0.072	1.841
LMO	Li1	8a	1.003	1.513
	Mn1	16d	0.994	0.828
	O1	32e	1.000	1.257

Supplementary References

1. Yoon, W. *et al*. In situ X-ray absorption spectroscopic study on $LiNi_{0.5}Mn_{0.5}O_2$ cathode material during electrochemical cycling." *Chem. Mater.* **15,** 3161-3169 (2003).
2. Balasubramanian, M. *et al*. In situ X‐ray absorption studies of a high‐rate $LiNi_{0.85}Co_{0.15}O_2$ cathode material. *J. Electrochem. Soc.* **147**, 2903-2909 (2000).
3. Hy, S. *et al*. Performance and design considerations for lithium excess layered oxide positive electrode materials for lithium ion batteries. *Energy Environ. Sci*. **9**, 1931-1954 (2016).
4. Tang, D. *et al*. Surface structure evolution of $LiMn_2O_4$ cathode material upon charge/discharge. *Chem. Mater.* **26,** 3535-3543 (2014).
5. Mao, C. *et al*. Selecting the best graphite for long-life, high-energy Li-ion batteries. *J. Electrochem. Soc.* **165**, A1837-A1845 (2018).
6. Zhan, C. *et al.* Mn (II) deposition on anodes and its effects on capacity fade in spinel lithium manganate–carbon systems. *Nat. Commun.* **4**, 2437 (2013).
